# Injectable hybrid hydrogels enable enhanced combination chemotherapy and roused anti-tumor immunity in the synergistic treatment of pancreatic ductal adenocarcinoma

**DOI:** 10.1186/s12951-024-02646-7

**Published:** 2024-06-20

**Authors:** Hao Zhou, Wei Wang, Zedong Cai, Zhou-Yan Jia, Yu-Yao Li, Wei He, Chen Li, Bang-Le Zhang

**Affiliations:** 1 Department of Pharmaceutics, School of Pharmacy, Fourth Military Medical University, Xi’an, 710032 China; 2 Key Laboratory of Pharmacology of the State Administration of Traditional Chinese Medicine, Fourth Military Medical University, Xi’an, 710032 China; 3 Department of Chemistry, School of Pharmacy, Fourth Military Medical University, Xi’an, 710032 China

**Keywords:** Pancreatic ductal adenocarcinoma, Injectable hydrogels, Ferroptosis, Tumor stroma, Anti-tumor immunity

## Abstract

Chemotherapy and immunotherapy have shown no significant outcome for unresectable pancreatic ductal adenocarcinoma (PDAC). Multi-drug combination therapy has become a consensus in clinical trials to explore how to arouse anti-tumor immunity and meanwhile overcome the poorly tumoricidal effect and the stroma barrier that greatly hinders drug penetration. To address this challenge, a comprehensive strategy is proposed to fully utilize both the ferroptotic vulnerability of PDAC to potently irritate anti-tumor immunity and the desmoplasia-associated focal adhesion kinase (FAK) to wholly improve the immunosuppressive microenvironment via sustained release of drugs in an injectable hydrogel for increasing drug penetration in tumor location and averting systematic toxicity. The injectable hydrogel ED-M@CS/MC is hybridized with micelles loaded with erastin that exclusively induces ferroptosis and a FAK inhibitor defactinib for inhibiting stroma formation, and achieves sustained release of the drugs for up to 12 days. With only a single intratumoral injection, the combination treatment with erastin and defactinib produces further anti-tumor performance both in xenograft and Kras^G12D^-engineered primary PDAC mice and synergistically promotes the infiltration of CD8^+^ cytotoxic T cells and the reduction of type II macrophages. The findings may provide a novel promising strategy for the clinical treatment of PDAC.

**Supplementary Information:**

The online version contains supplementary material available at 10.1186/s12951-024-02646-7.

## Introduction

Pancreatic ductal adenocarcinoma (PDAC) accounts for over 80% of all kinds of pancreatic cancer and it is known as one type of terribly malignant tumor due to the extremely short survival of less than 1 year [1, 2]. Chemotherapy and various chemotherapeutic combinations have been implemented in clinical trials for unresectable PDAC that accounts for over 50% in all PDAC stages, but patients still have no apparent benefit [3]. Anti-tumor immunity has become the most promising treatment strategy for unresectable PDAC, and the multi-drug synergism for arousing anti-tumor immune responses has become a consensus for PDAC treatment [4]. Many clinical trials have been conducted to find an effective combination such as NCT03193190, NCT03184870, NCT04060342, NCT03214250, NCT04787991, etc., but the unsatisfactory outcome may be attributed to the limited release of neoantigens by chemotherapy and the special tumor microenvironment of PDAC [5].

Ferroptosis, a pro-inflammatory programmed cell death driven by the accumulation of iron-dependent peroxidative polyunsaturated fatty acids in cell membranes, has attracted great attention as a potential anti-tumor strategy for years [6]. However, few clinical trials were conducted for cancer therapy utilizing ferroptosis, presumably resulting from the poor pharmacokinetic characteristics, required high-dose administration that would lead to serious systematic side effects, and little penetration in solid tumors. Recently, the ferroptosis of cancer cells has been found to be potent in irritating innate and adaptive anti-tumor immunity. Ferroptotic dying cancer cells exhibit the capacity to release chemokines such as CXCL1, CCL2, and CXCL10 that recruit neutrophils, releasing immunogenic DAMPs as the “find me” and “eat me” signals recognized by antigen presenting cells, and releasing immunogenic signals such as HMGB1 to activate memory CD4^+^ T cells and to induce the maturation of bone marrow dendritic cells [7–10]. Intriguingly, the role of ferroptosis in different types of cancer varies with different metabolisms [11–14]. What is more important, due to the PDAC property of both excessive uptake of glutamate transformed from proline and limited uptake of glucose uptake, PDAC exhibits a remarkable susceptibility to selective ferroptosis [11]. Those suggest that ferroptosis remains a promising tool for regulating anti-tumor immunity and a probably feasible strategy to achieve tumoricidal effect specifically in PDAC.

In addition to the particular metabolic vulnerability in PDAC, the ineffectiveness of PDAC treatment is partly because of an immunosuppressive tumor microenvironment (TME), which is closely related to pancreatic tumor stroma [15]. The abundant stroma of PDAC accounts for up to 80% of the tumor mass, which is widely considered to consist of tumor-associated fibrocytes, and acellular components such as collagens, fibronectin, and hyaluronic acid [16, 17]. The tumor stroma ultimately results in desmoplasia and fibrotic responses creating a physical barrier that insulates tumors from drugs and hinders the infiltration of immune cells into tumors [18, 19]. Several stroma-depleting therapeutic strategies have been attempted such as using PEGylated hyaluronidase (PEGPH20) (HALO-109-301, NCT02715804) to degrade hyaluronic acid or using marimastat to inactivate matrix metalloproteinases [20], but no significant benefit was shown in progression-free survival (PFS) or overall survival (OS) compared with the first-line chemotherapy [12, 21, 22]. In addition, several kinases such as Rho-associated protein kinases (ROCKs), focal adhesion kinases (FAKs), and discoidin domain receptor 1 (DDR1) were found to play important roles in stroma production [13]. Some small molecules targeting these kinases such as fasudil, defactinib, and 7rh exhibited the capacity of inhibiting stroma formation and show some promise for PDAC therapy [12, 23]. Of note were FAK over-expression and activation, as a crucial feature in the majority of human PDAC epithelia, to be a principal driver in PDAC desmoplasia and the generation of an immunosuppressive TME [21], since intratumoral regulatory T cells (Treg) were up-regulated by FAK-dependent expression of CC-chemokine ligand 5 (CCL5) and the cytokine transforming growth factor β2 (TGF-β2) [24], the FAK inhibitor defactinib (also known as VS-6063) has been shown to inhibit FAK overexpression and effectively reduce the formation of tumor stroma in PDAC [25]. Defactinib has been enrolled in clinical trials for treatments of several kinds of cancer (NCT01870609, NCT02546531, NCT02758587), but a phase II clinical trial found no improvement in PFS or OS in malignant pleural mesothelioma [26]. Those suggested FAK inhibitors need further improvement for anti-tumor therapy probably by combining with other chemotherapeutic strategies and drug delivery techniques to increase the outcome.

Additionally, a common obstacle to cancer therapy is systemic side effects resulting from non-specific drug distribution in tissues, especially in PDAC due to the stroma barrier [27]. Various drug delivery systems were developed to increase drug accumulation in tumor sites, however, toxicity to the liver, kidney, and other tissues via intravenous administration still can not be ignored [28]. Injectable hydrogels as a novel delivery strategy can bring the solution through sol-gel transition since they have the advantage of tunable drug release properties, protection of labile drugs from degradation, and manageable degradability [29]. In recent years, the minimally invasive technique using injectable hydrogels has developed in clinical treatments and makes possible localized drug delivery and release directly into tumors in situ [30]. As for unresectable PDAC, the minimally invasive technique is recommended in clinics for local drug delivery. The ideal in situ injectable hydrogel needs prolonged retention in vivo. However, weak hydrogels formed by biocompatible materials approved by the US Food and Drug Administration usually dissolve rapidly. Moreover, chemical modifications designed to enhance cross-linking would add an additional regulatory burden [31]. Traditional hydrogels for free drugs often have poor encapsulation and burst release from hydrogels and rapid degradation in vivo, which limits further clinical application. More exploration is essential to obtain hydrogels with good physical structures and rheological and mechanical properties.

**Scheme 1:**
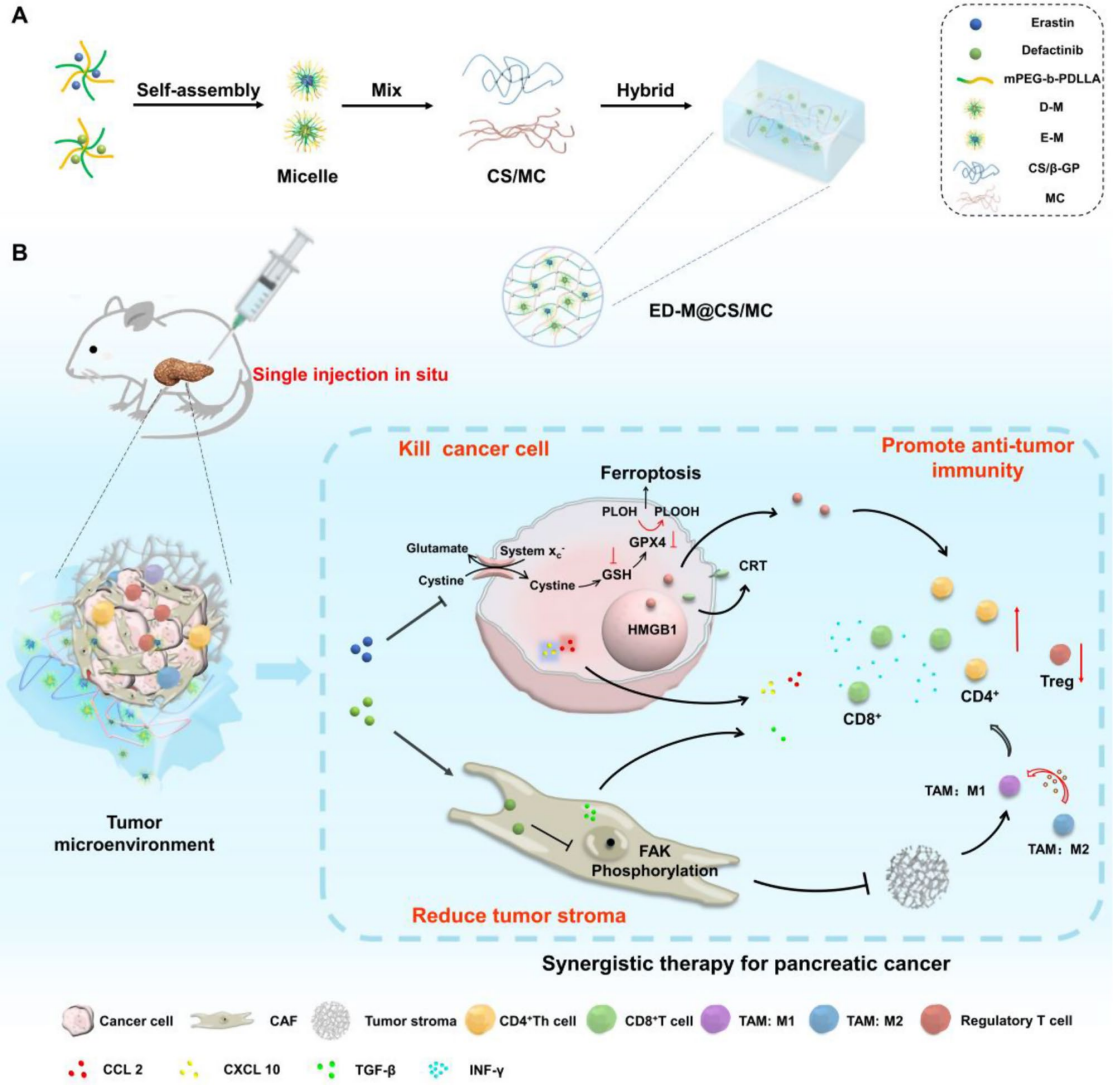
The construction and therapeutic strategy of ED-M@CS/MC for PDAC. (**A**) Preparation of the injectable hydrogel ED-M@CS/MC. (**B**) The combination therapeutic strategy through ferroptosis and stromal modulation

In this study, inspired by both ferroptosis and FAK inhibitors irritating anti-tumor immune responses, a sustainable released hybrid hydrogel ED-M@CS/MC, which combined the therapeutic strategies of ferroptosis and stromal modulation for synergistic immunotherapy was designed for the combination treatment of PDAC (Scheme 1). The ED-M@CS/MC hydrogels were composed of chitosan (CS) and methylcellulose (MC) and hybridized the micelles of erastin (E), a canonical ferroptosis inducer, and defactinib (D), a FAK inhibitor for inhibiting tumor stroma formation. Among them, CS is non-irritating polymeric polysaccharides most widely used in pharmaceuticals [32], MC is a cellulose derivative [33]which is used to improve intratumoral retention when incorporated with CS to form the hydrogel. The introduction of micelles not only allows for the encapsulation of these two poorly soluble drugs but also further controls the release and extends the retention time of the drugs in the tumor tissue. Hydrogel incorporating drug-loaded micelles can further extend the sustained and long-term effect of erastin or defactinib released from hydrogel. Erastin micelles (E-M) and defactinib micelles (D-M) are prepared respectively with mPEG_2000_-b-PDLLA_2000_ and incorporated into the CS/MC hydrogel to obtain ED-M@CS/MC (Scheme 1A). Due to the controllable release profiles of ED-M@CS/MC, through only single intratumoral injection, erastin and defactinib can be sustainably released from the micelle in hydrogel and play a sustainably synergistic therapeutic role. Erastin can induce ferroptosis to kill the tumor cells and explore the metabolic vulnerability in PDAC [34, 35]. Defactinib can inhibit FAK phosphorylation to ameliorate desmoplasia and reduce the formation of tumor stroma, thus synergistically facilitating the infiltration of cytotoxic T lymphocytes and decreasing Treg and M2 type macrophages. Due to the combinated anti-tumor performances of ED-M@CS/MC involved both the chemotherapy and the immunological effect based on improving anti-tumoral immune infiltration and immunogenic DAMPs, C57BL/6 mice and *Kras*^*LSL−G12D/+*^*(KI/+), Trp53*^*LSL − R172H/+*^*(KI/+)*, and *Pdx1-Cre (TG/+)* (KPC) mice obtained through *Kras*^*LSL−G12D/+*^*(KI/+), Trp53*^*LSL − R172H/+*^*(KI/+)* (KP) mice and *Pdx1-Cre (TG/+)* (PC) mice were used to investigate the synergistic combination effect of chemotherapy and anti-tumor immunity treatment. The constructed injectable hybrid ED-M@CS/MC hydrogels for synergistically combined strategy show beneficial anti-tumor performance both in xenograft and Kras^G12D^-engineered primary PDAC mice.

## Materials and methods

### Materials

Defactinib, erastin, methylcellulose (MW: 15 mPa·s) and β-sodium glycerophosphate (β-GP) were purchased from Aladdin Biochemical Technology Co., Ltd. (Shanghai, China). mPEG_2000_-b-PDLLA_2000_ and lipo-Cy3(Me) acid (Cy3) were purchased from YUSI Pharmaceutical Technology Co., Ltd. (Chongqing, China). Chitosan (MW: 50–190 KDa) was purchased from Sigma-Aldrich, Inc. (St. Louis, MO, U.S.A.). Sulfo-Cyanine5.5 (Cy5.5) was purchased from MedChemExpress (Monmouth Junction, NJ, U.S.A). Acetic acid and dimethyl sulfoxide (DMSO) were purchased from KeHao Biological Technology (Xi’an, China). Dulbecco’s modified eagle medium (DMEM) and FBS were purchased from ExCell Bio Co., Ltd. (Shanghai, China). Matrigel was purchased from Corning, Inc. (Corning, NY, USA). TUNEL cell apoptosis detection kit, glutathione detection kit and MDA detection kit, Masson trichrome kit, Sirius red staining kit were purchased from Servicebio Technology Co., Ltd. (Wuhan, China). C11-BODIPY^581/591^ probe was purchased from Glpbio Technology Co., Ltd. (Guangzhou, China). All antibodies for immunohistochemistry and immunofluorescence were purchased from Abcam (Cambridge, MA, U.S.A) and used as received. Tumor dissociation kit (Mouse) was purchased from Miltenyi Biotec GmbH (Bergisch Gladbach, Germany). All antibodies for flow cytometry were purchased from BioLegend (San Diego, CA, U.S.A) and used as received. eBioscience™ Mouse Regulatory T Cell Staining Kit ^#1^ was purchased from Thermo Fisher Scientific, Inc. (Waltham, MA, U.S.A). OptiLyse C lysing solution were purchased from Beckman Coulter (Brea, CA, U.S.A). HMGB1, IFN-γ kit, and CXCL10 ELISA kits were purchased from Thermo Fisher Scientific, Inc. (Waltham, MA, U.S.A). ATP assay kit was purchased from BioVision (Milpitas, CA).

### Cells and animals

Panc02 cells were purchased from Procell Life Science & Technology Co., Ltd. (Wuhan, China). Panc02 Firefly Luciferase cells were purchased from the CytoBiotech (Xi’an, China). Cells were cultured in DMEM containing 1% antibiotics and 10% FBS in an incubator at 37 °C in 5% CO_2_. C57BL/6 mice (18–20 g in body weight, 7–8 weeks) were supplied by the Laboratory Animal Center of the Fourth Military Medical University (Xi’an, China). *Kras*^*LSL−G12D/+*^*(KI/+), Trp53*^*LSL − R172H/+*^*(KI/+)*, and *Pdx1-Cre (TG/+)* (KPC) mice have been obtained through KP mice and PC mice as the method reported previously [36]. KP mice and PC mice were obtained from Cyagen Biosciences (Suzhou, China). *Kras*^*LSL−G12D/+*^*(KI/+), Trp53*^*LSL − R172H/+*^*(KI/+)* (KP), and *Pdx1-Cre (TG/+)* (PC) mice are syngeneic healthy littermates generated during routine breeding of KPC mice. All animal experiments were admitted by the Animal Ethics Committee of Fourth Military Medical University (IACUC-20,231,241, IACUC-20,231,242).

### Preparation and characterization of the micelles of E-M and D-M

E-M and D-M micelles were prepared using the thin-film hydration method. Briefly, mPEG_2000_-b-PDLLA_2000_ (50.0 mg), defactinib (4.5 mg), or erastin (4.5 mg) were respectively weighed and completely dissolved in chloroform (3 mL) in the round flask. The chloroform was removed by rotary evaporation at 55 rpm for 5 min at 35 °C until a thin film formed, which was then vacuum-dried overnight. Subsequently, the dried film was hydrated under normal pressure at 37 °C with gentle agitation on a tabletop rocker operating at 55 rpm for 5 min to obtain the D-M or E-M micellar solutions. Blank micelles (M) were prepared following the same procedure. Particle size and polydispersity index (PDI) of D-M and E-M were measured using dynamic light scattering (Nano series ZSE, Malvern, U.K.). Transmission electron microscopy (TEM) (Talos F200X, Thermo Fisher Scientific, U.S.A) was employed to observe the morphology of D-M and E-M after staining them with 2% phosphotungstic acid for 2 min and removing the remaining liquid using filter paper before imaging. The drug loading capacity and encapsulation efficiency of D-M and E-M were determined via UV-vis spectrophotometry (UV-3200, MAPADA, China) after dissolving lyophilized samples in DMSO.

### Preparation of injectable ED-M@CS/MC hydrogels

Chitosan was exposed to ultraviolet light overnight to achieve sterilization. A chitosan solution was obtained by dissolving 2.5% (w/v) in 0.5% (v/v) acetic acid with stirring. A β-GP solution of 56% (w/v) in purified water was sterilized using a 0.22 μm filter at 4 °C. Subsequently, the β-GP was slowly added to the chitosan solution under magnetic stirring to obtain a homogeneous and clear CS/β-GP solution. A methylcellulose solution of 20% (w/v) was prepared by dissolving in cold purified water. A solution containing equal amounts of E and D was prepared by mixing solutions of D-M and E-M, and then this solution was mixed with the methylcellulose stock solution and the CS/β-GP solutions at a volume ratio of 1:1:2 with stirring to obtain injectable ED-M@CS/MC hydrogel solution. Gelation generally occurs at a temperature of 37 °C. The method for preparing D-M@CS/MC, E-M@CS/MC, M@CS/MC, and Cy3-M@CS/MC hydrogels followed that used for ED-M@CS/MC and blank micelles replaced either E-M, D-M or Cy3-M respectively.

### The morphology of ED-M@CS/MC hydrogels

The morphology of the ED-M@CS/MC was characterized using Cryo-SEM (FEI Quanta 450, Japan, Quorum PP3000T, U.K.). ED-M@CS/MC was prepared in 12 well plates and gelled at 37 °C, then conductive carbon glue was placed on the sample platform and carefully fix the specimen with tweezers. The sample platform and attached specimens are rapidly frozen in a liquid nitrogen mixture for 30 s and then transferred to a vacuum-sealed sample preparation room through a low-temperature frozen preparation transmission system. Subsequently, sublimation and gold plating are performed: the specimens undergo sublimation for 10 min at -90 °C, followed by metal spraying for 60 s at a current of 10 mA. The sample was sent to the SEM sample room for observation, with a cooling plate temperature of -140 °C and an acceleration voltage of 10 KV.

### Rheological properties of ED-M@CS/MC

Rheological properties were analyzed using a rotational rheometer (Mars40, HAAKE, Germany) equipped with a stainless-steel parallel plate measuring system (25 mm plate diameter). The sample was placed in the center of the bottom parallel plate, and the top plate was moved to the measuring position (a 1 mm gap size was used). Afterward, the sample was trimmed using a spatula such that the sample edge was approximately flush with the top parallel plate. Monitoring variation in storage modulus (G′), and loss modulus (G″) under a constant shear stress of 1 Pa and frequency of 1.0 Hz. The gelation point was considered to be the point at which G′ and G″ were the same value. For viscosity measurement, the controlled frequency with 1 rad s^− 1^ and the changeable strains from 0.01 to 100% were used.

### Determination of sol-gel transition time

The sol-gel transition time was tested by a tube-inverting method whereby vials containing 0.5 mL of freshly prepared pre-gel solution were immersed in a water bath set at a temperature of 37 °C until gelation occurred. Gelation time was recorded when the liquid ceased flowing after horizontal inversion every half minute. The sol-gel transition time was shown as the time at which the flow of the systems stopped.

### In vitro release behavior of hydrogels

200 µL Cy3-M@CS/MC hydrogel solution was added to a dialysis bag with a molecular weight cut-off of 3500 Da and incubated at 37 °C to form a gel. After immersed in conical flasks containing 20 mL phosphate buffer release medium (pH 7.4) containing Tween-80 (1.0 wt%), the release behavior of hydrogels was studied in a constant temperature shaking incubator (37 °C, 80 rpm). 2 mL of the release solution outside the dialysis bag was taken out at predetermined time points (12 h, 24 h, 36 h, 2 d, 3 d, 4 d, 5 d, 7 d, 9 d, 12 d) and 2 mL of fresh phosphate buffer was added. The amount of released Cy3 in the supernatant was measured by a microplate reader (SpectraMax iD3, Molecular Devices, USA), and the cumulative release of Cy3 from Cy3-M@CS/MC hydrogels was calculated.

### In vivo release behavior of hydrogels

The release behavior in vivo was determined after the injection of Cy3-M@CS/MC hydrogels in the back of C57BL/6 mice. 200 µL Cy3-M@CS/MC hydrogels were injected into the back of the mice using 29G needle syringe. The fluorescence imaging was performed on a small animal in vivo imaging instrument (PerkinElmer, Waltham, MA, U.S.A) at predetermined time points (0 d, 3 d, 6 d, 9 d, 12 d).

### Degradation profiles in vivo

The degradation profile was determined in vivo after the injection of hydrogels in the back of C57BL/6 mice. 200 µL M@CS/MC hydrogels were injected into the dorsal region of C57BL/6 mice using 29G needle syringe. The degradation was monitored by quantifying the volume and the weight of the remaining M@CS/MC hydrogels at specified time intervals (0 d, 3 d, 6 d, 9 d, 12 d) following its administration into the body. The hydrogels volume was measured using a caliper and calculated as length × width^2^/2 every 3 days.

### Biocompatibility in vivo

Healthy C57BL/6 mice were used for testing the biocompatibility of the hydrogels. Specifically, 200 µL of M@CS/MC hydrogels was injected into the pancreatic region using 29G needle syringe under sterile conditions. 200 µL of PBS served as the control. Mice were monitored daily for 12 days and euthanasia was performed on the mice for subsequent H&E staining to analyze the pathological changes in vital organs and subcutaneous tissue. And the blood was collected and the supernatant serum was taken by centrifugation at 3000 rpm for 15 min at 4 °C, and the concentrations of alanine aminotransferase (ALT), aspartate aminotransferase (AST), blood urea nitrogen (BUN) and creatinine (CREA) were detected by the blood biochemical analyzer.

### Anti-tumor effect and safety evaluation in xenograft and Kras^G12D^-engineering mice

The synergistic anti-tumor effect by the combination therapy using injectable hybrid hydrogels involved the performances both the chemotherapy and the immunological effect, so C57BL/6 mice were used. The mixture of 100 µL cell suspension containing Panc02 cells at a density of 2 × 10^7^ cells/mL and 100 µL Matrigel was injected into the right flank regions of the C57BL/6 mice to establish the xenograft animal model of pancreatic cancer. Once the tumor volume reached 100 mm^3^, the mice were randomly divided into four groups (*n* = 5), including M@CS/MC (control), D-M@CS/MC (1 mg kg^− 1^ of defactinib) and E-M@CS/MC (1 mg kg^− 1^ of erastin), and ED-M@CS/MC (1 mg kg^− 1^ of erastin and 1 mg kg^− 1^ of defactinib). The mouse were fixed in a supine position and an intratumoral injection was performed using 29G needle syringe into tumor sites very carefully to avoid bleeding. The tumor volume was measured using a caliper and calculated as length × width^2^/2 every 2 days. The body weight was monitored every two days. The tumor growth was also observed at days 0, and 12 through in vivo bioluminescence imaging spectrum system (IVIS). After 12 days, the blood was collected and the supernatant serum was taken by centrifugation at 3000 rpm for 15 min at 4 °C, and the concentrations of alanine aminotransferase (ALT), aspartate aminotransferase (AST), blood urea nitrogen (BUN), and creatinine (CREA) were detected by the blood biochemical analyzer (Chemray 800, Shenzhen Leidu Life Technology, China). Euthanasia was performed on the mice of each group and the organs including the heart, lung, liver, spleen, and kidney underwent paraffin embedding for hematoxylin-eosin staining. Tumor tissues were excised, weighed, and photographed and part of each tumor was stored at -80 °C or fixed in formalin for the further analysis.

The animal model of spontaneous pancreatic cancer was established by Kras^G12D^-engineering mice. Genetic identification of the *Kras*^*LSL−G12D/+*^*(KI/+), Trp53*^*LSL − R172H/+*^*(KI/+)*, and *Pdx1-Cre (TG/+)* (KPC) mice was conducted through tail DNA genotyping. KPC mice can spontaneously develop pancreatic cancer when they reach 16 weeks old. Pancreatic tumors in KPC mice were assessed using a small animal ultrasound imaging system (Vevo 2100, Visual Sonics, Canada). Once obvious tumors were observed in the pancreas using the ultrasound imaging system, the mice were randomly divided into four groups (*n* = 5), including M@CS/MC (control), D-M@CS/MC (1 mg kg^− 1^ of defactinib) and E-M@CS/MC (1 mg kg^− 1^ of erastin), and ED-M@CS/MC (1 mg kg^− 1^ of erastin and 1 mg kg^− 1^ of defactinib). Intratumoral injection was performed. Ultrasound imaging was conducted on days 0, 6, and 12 to monitor tumor changes. The body weight was monitored every two days. After 12 days, the blood was collected and the supernatant serum was taken by centrifugation at 3000 rpm for 15 min at 4 °C, and the concentrations of alanine aminotransferase (ALT), aspartate aminotransferase (AST), blood urea nitrogen (BUN) and creatinine (CREA) were detected by the blood biochemical analyzer. The organs including the heart, lung, liver, spleen, and kidney underwent paraffin embedding for hematoxylin-eosin staining. The pancreatic tumors were excised for weighing and photography. The portion of each tumor tissue was stored at -80 °C or fixed in formalin for further analysis. Survival analysis was performed in each group (*n* = 4) starting from the time of mouse birth.

### H&E staining and TUNEL staining

The tumor tissues of xenograft or Kras^G12D^-engineering mice in each group were kept in 4% natural buffer formalin at 4 °C for 24 h before they were paraffin-embedded. The slides were deparaffinized, soaked in xylene for 3 min, xylene/ethanol (1:1 v/v) for 3 min, absolute ethanol for 3 min, 95% ethanol for 3 min, 70% ethanol for 3 min, and 50% ethanol for 3 min, and then rinsed with tap water. Nuclei blue fluorescent staining was performed using NucBlue Fixed Cell ready probes that were added for 10 min followed by rinsing with tap water. The samples were cut into 4 μm slides. The sections were then stained with H&E as instructed. The cell death analysis was performed using the TUNEL kit as instructed by the protocol.

### Immunofluorescence analysis

The above samples were cut into 4 μm slides. The slides were blocked with 5% bovine serum albumin and then incubated with the primary antibodies (anti-Ki67, 1:300; anti-cytokeratin 19, 1:100; anti-xCT, 1:100; anti-glutathione peroxidase 4,1:100; anti-α-smooth muscle actin, 1:400; anti-FAK, 1:100; anti-FAK (phospho Y397), 1:100; anti-calreticulin; anti -HMGB1, 1:200; anti-CD206, 1:1500;) overnight at 4 °C. After three washes with PBS, the samples were incubated with the secondary antibody (Cy3-goat anti-rabbit, 1:300; 488-goat anti-rabbit, 1:300) for 2 h at room temperature. All slices were observed under an inverted fluorescence microscope (Nikon Eclipse C1, Japan) and the slices were digitally scanned using a high-resolution slice scanner (Pannoramic 250FLASH, 3DHistech, Hungary).

### Lipid peroxidation measurement

The fluorescent probe C11-BODIPY^581/591^ was used to evaluate the lipid peroxidation. All subsequent steps were followed as instructed by the manufacturer.

### GSH and MDA measurement

Malondialdehyde (MDA) concentrations were measured with a malondialdehyde test kit by using the thiobarbituric acid reactive substance method, and glutathione (GSH) concentrations were determined using a reduced GSH assay kit. All the steps were followed as instructed by the manufacturer.

### Immunohistochemistry analysis

For paraffin-embedded sections, DAB IHC was employed. The tumor tissue slices were stained using a masson trichrome kit, and sirius red staining kit to detect the collagen following the manufacturer’s instructions.

### Tumor-infiltrating immune cell detection by FCM

The weighed tumor tissues were minced and digested in a 2.5 mL mixture containing 100 µL of enzyme D, 10 µL of enzyme R, and 12.5 µL of enzyme A derived from the tumor dissociation kit (Mouse) in DMEM medium using a tissue cell suspension preparation apparatus (Miltenyi Biotec GentleMACS Dissociator with heaters, German) at 37 °C for 1 h. Debris was removed by filtration through a 40 μm mesh, and the red blood cells were removed using a red blood cell lysis buffer. The mixture was washed with PBS before further analysis.

The CD3^+^CD4^+^ T cells, CD3^+^CD8^+^ T cells, CD4^+^CD25^+^Foxp3^+^ regulatory T cells, M1 macrophages and M2 macrophages were stained with fluorescence-labeled antibodies, CD11b (clone M1/70), CD206 (clone C068C2), F4/80 (clone BM8), CD4 (clone GK1.5), CD8 (clone 53–5.8), Foxp3 (clone MF-14) following the manufacturer’s instructions. Intracellular staining was performed after fixation and permeabilization with eBioscience™ mouse regulatory T cell staining kit. Data analysis was performed using FlowJo software (FlowJo v10.8.1, Ashland).

### Cytokines and ATP assay

Tumor tissues were minced and grinded with a tissue cell suspension preparation apparatus in PBS buffer. Debris was removed by filtration through a 40 μm mesh, and the single-cell suspension was collected and centrifuged at 1000 rpm to obtain the supernatant of interstitial fluid [37, 38]. According to the manufactures’ instruction, the level of secreted HMGB1, IFN-γ, and CXCL10 was detected using HMGB1, IFN-γ, and CXCL10 ELISA kits. In addition, the level of ATP was tested by ATP assay kit.

### Statistics analysis

All results were presented as mean ± SD. The one-way analysis of variance (ANOVA) with Tukey’s post-hottest was used for multiple comparisons (when more than two groups were compared). The logrank test was used in survival analysis. All statistical analyses were carried out with the Prism software package (PRISM 8.0, GraphPad Software). *P* < 0.05, *P* < 0.01 and were represented for statistical difference and ns stands for not significant.

## Results and discussion

### Preparation and characterization of ED-M@CS/MC hydrogels

**Fig. 1:**
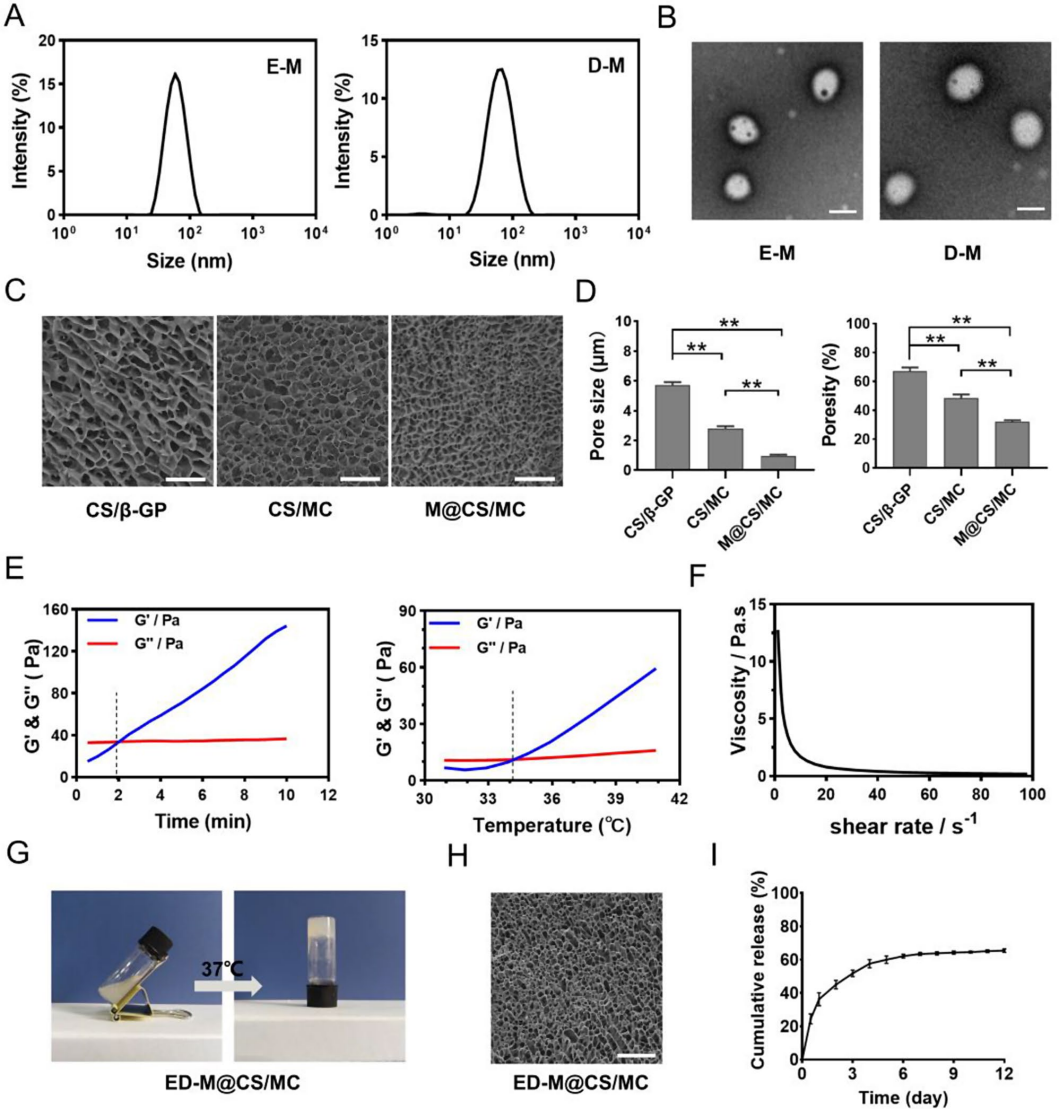
Characterization of E-M, D-M and ED-M@CS/MC hydrogels. (**A**) DLS size measurement of E-M, D-M. (**B**) TEM image of the morphology of E-M and D-M. Scale bar: 50 nm. (**C**) Cryo-SEM image of the inner porous structures of CS/GP, CS/MC, M@CS/MC, scale bar: 40 μm. (**D**) Pore size and porosity of CS/GP, CS/MC, M@CS/MC hydrogels (*n* = 3). (**E**) Rheology properties of ED-M@CS/MC (the storage modulus: G′, the loss modulus: G″). (**F**) Viscosity of ED-M@CS/MC solution (*n* = 3). (**G**) Images of ED-M@CS/MC transition from sol to gel state. (**H**) Cryo-SEM image of the ED-M@CS/MC hydrogels. Scale bar: 40 μm. (**I**) The cumulative release of Cy3-M@CS/MC hydrogels (*n* = 3)

Due to the presence of hydrophilic groups in the structure of traditional hydrogels, the insoluble drugs erastin (E) and defactinib (D) are hardly dispersed or released. To address this issue, by using mPEG_2000_-b-PDLLA_2000_, the widely used block copolymer for forming micelles [39], erastin and defactinib were conveniently incorporated into micelles, named E-M and D-M respectively. The dynamic light scattering (DLS) analysis revealed that the particle size of E-M and D-M were 63 nm and 68 nm, respectively (Fig. 1A), and both the values of PDI were less than 0.3. Transmission electron microscopy (TEM) images showed that E-M and D-M had a homogeneous spherical morphology, and the particle size was consistent with that measured by DLS (Fig. 1B). In general, in situ injection of a solution could transude from the injection site and randomly migrate, leading to limited clinical application, whereas in situ injection of hydrogels mitigates this issue [40]. Chitosan (CS), a most plentiful natural polysaccharide, has valuable characteristics of biocompatibility and biodegradability [41]. We utilized chitosan as hydrogels material to cross-link with β-sodium glycerophosphate (β-GP). It was found that the gelating process and subcutaneous degradation of the chitosan hydrogel exhibited a long gelating time of over 8 min and rapid degradation within 4 days (Table S1, Fig. S1). As known, the clinical application of hydrogels is hindered by short retention time in vivo [42]. To improve the sol-gel transition properties of the CS/β-GP hydrogels, the polymeric methylcellulose (MC) was added to improve the thermal gelation properties via the hydrogen bond and hydrophobic interactions [43, 44]. When incorporating MC, a significant reduction in gelling time was observed (Table S2) and the degradation time was extended to more than 12 days (Fig. S2). The composition of the E-M@CS/MC, D-M@CS/MC and ED-M@CS/MC was summarized in Table S3. The cryo-SEM images showed that CS/MC exhibited a smaller pore size, denser structure, and decreased porosity compared to CS/β-GP (Fig. 1C, D). This observation followed the principles of sufficient cross-linking density making hydrogels more resistant to deformation or disintegration [45]. The porous nature of hydrogels enables incorporating particles smaller than the existing pores [40, 46], implying strengthened intermolecular forces of loading micelles resulted in more compact and denser hydrogels, probably helpful for sustained drug release [46]. The injectable properties of ED-M@CS/MC hydrogels are crucial for the intratumoral injection. As shown in Fig. 1E-G, the gelating process of ED-M@CS/MC hydrogels occurred at 1 min 49 s at 34 °C (Fig. 1E). The viscosity of the ED-M@CS/MC hydrogels were examined using consecutive flow measurements (Fig. 1F). The viscosity reduced with incremental shear rate, showing shear-thinning property. The thermosensitive sol-gel transition property of ED-M@CS/MC at 37 °C was also observed by the tube inverting test (Fig. 1G). Cryo-SEM images of ED-M@CS/MC showed a typical porous morphology of ED-M@CS/MC in consistence with that of M@CS/MC (Fig. 1H). In vitro drug release behavior of the hydrogels was conducted and showed the cumulative release of 63.8% on the 12th day (Fig. 1I). The drug release behavior was also assessed in vivo by imaging system. The results showed that the fluorescent signal in Cy3-M@CS/MC hydrogels decayed with time and could last for 12 days (Fig. S3). When observed the in vivo degradation of the gel, it was determined that the gel has a minimum residence time of 12 days within the body (Fig. S4). To evaluate the toxicity of the hydrogels, the histological examination of the major organs in mice was performed. No obvious pathological change was observed in each group (Fig. S5).

### Combination treatment of pancreatic cancer through ferroptosis and stromal modulation in xenograft mice

#### The anti-tumor efficacy of ED-M@CS/MC injectable hydrogel in xenograft PDAC model

**Fig. 2:**
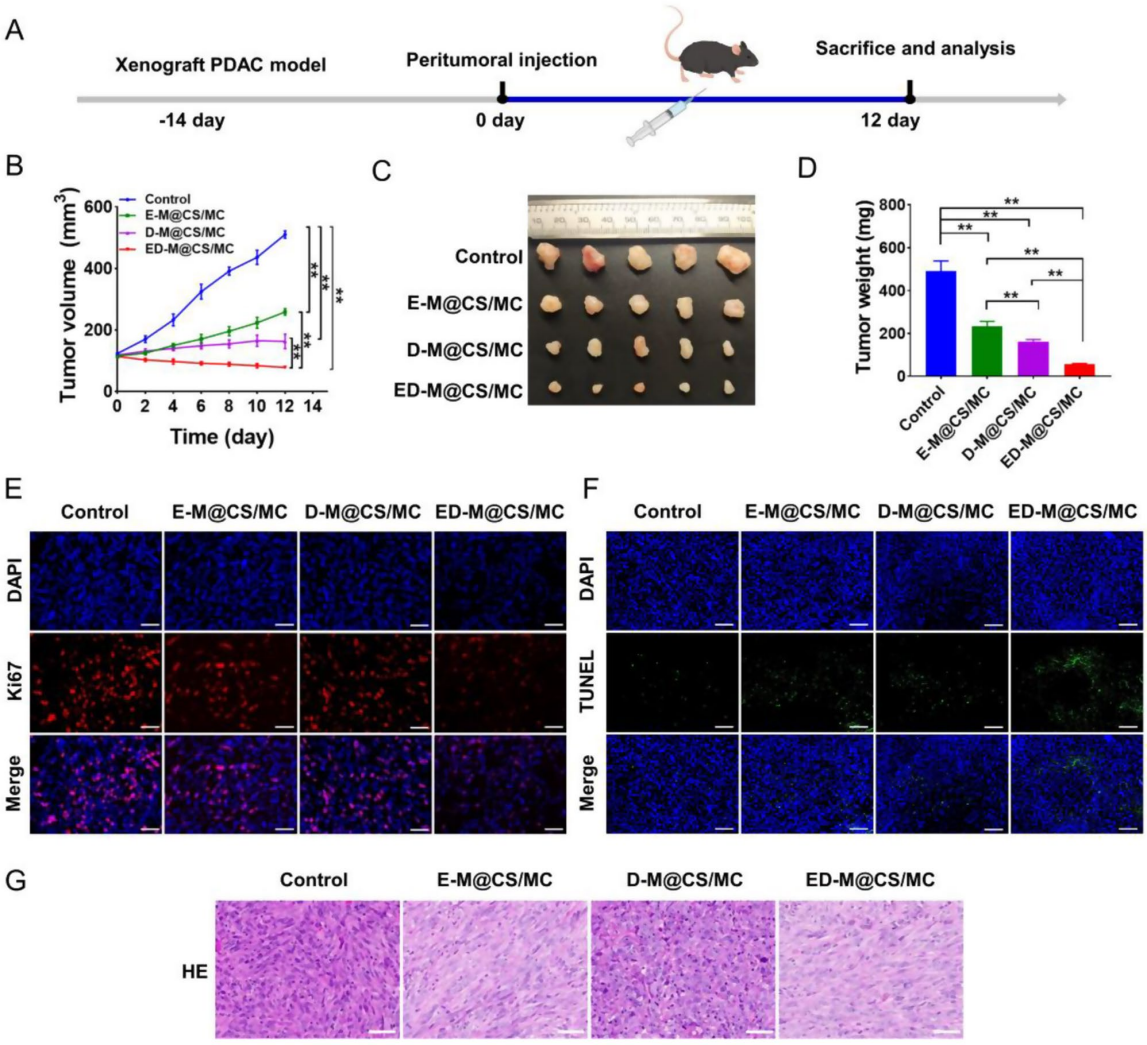
Anti-tumor efficacy in Panc02-bearing xenograft mice. (**A**) Schedule for model establishment, drug treatment and tissue collection and analysis. (**B**) Tumor volumes (*n* = 5, Mean ± SD, **P < 0.01). (**C**) Images of the tumors after different treatments. (**D**) Tumor weights after different treatments (*n* = 5, Mean ± SD, **P < 0.01). (**E**) Representative images of Ki67 expression tested by immunofluorescence staining. Scale bar: 50 μm. (**F**) Representative images of TUNEL staining. Scale bar: 50 μm. (**G**) H&E staining analysis. Scale bar: 50 μm

Although PDAC exhibited a remarkable susceptibility to ferroptosis [11], the therapeutic effect of ferroptosis in PDAC remains insufficiently investigated. It is imperative to assess the potential efficacy of ferroptosis on pancreatic tumor cells. The anti-tumor effects of D-M@CS/MC, E-M@CS/MC and ED-M@CS/MC hydrogels were evaluated using the Panc02-bearing xenograft model after a single intratumoral injection (Fig. 2A). The tumor volumes were monitored and it showed that the E-M@CS/MC or D-M@CS/MC treatment groups resulted in 2.77 times or 3.15 times reduction in tumor volumes compared with the control, while the ED-M@CS/MC group achieved an impressive 9.15 times decrease in tumor volumes (Fig. 2B). The tumors were harvested and weighed on the 12th day in each group (Fig. 2C). It showed a significant reduction in the average tumor mass in the E-M@CS/MC or D-M@CS/MC group with values of 234 ± 22 mg or 160 ± 11 mg respectively compared with the control group of 490 ± 48 mg. Additionally, the ED-M@CS/MC treatment group exhibited an even further decrease in mass (56 ± 4 mg) compared with both the D-M@CS/MC and E-M@CS/MC groups, indicating a synergistic tumoricidal effect through erastin (E) and defactinib (D) (Fig. 2D). The tumor growth of the Panc02-luc-bearing mice was also observed using bioluminescence imaging, yielding consistent data at the treatment endpoint (Fig. S6).

The tumor proliferation was further assessed by examining the expression of Ki67, a well-documented marker for malignant proliferation that is involved in mitosis. A slight decrease in Ki67 expression was noted in the D-M@CS/MC group, as well as in the E-M@CS/MC group, when compared to the control group. Furthermore, treatment with ED-M@CS/MC significantly reduced the number of Ki67-positive cells compared to all other groups (Fig. 2E). TUNEL assay showed low fluorescence in all groups, indicating that neither erastin nor defactinib induced the formation of conventional apoptotic DNA fragmentation (Fig. 2F). Meanwhile, H&E staining showed the observed decrease in tumor cell density treatment with E-M@CS/MC and a significant reduction of tumor stroma when treated with D-M@CS/MC. The combination of treatments led to a significant decrease in both tumor cells confluence in the E-M@CS/MC group, and the combination of erastin and defactinib led to a further decrease in tumor cells (Fig. 2G). The body weight of mice was monitored throughout the experiments, and the major organs including the heart, liver, spleen, lung, and kidney were collected for histological examination using H&E staining. It showed non-significant changes in body weight between the treatment groups and control group and inconspicuous histological damage in the major organs (Fig. S7 and S8). The parameters indicating liver and kidney injury were also assessed, all of which fell within the normal reference ranges (Fig. S9). These findings revealed the favorable anti-tumor profile and negligible systemic toxicity of the erastin- or/and defactinib-loaded hydrogel in the treatment of PDAC.

### Ferroptotic tumoricidal activity of ED-M@CS/MC hydrogels in xenograft PDAC model

**Fig. 3:**
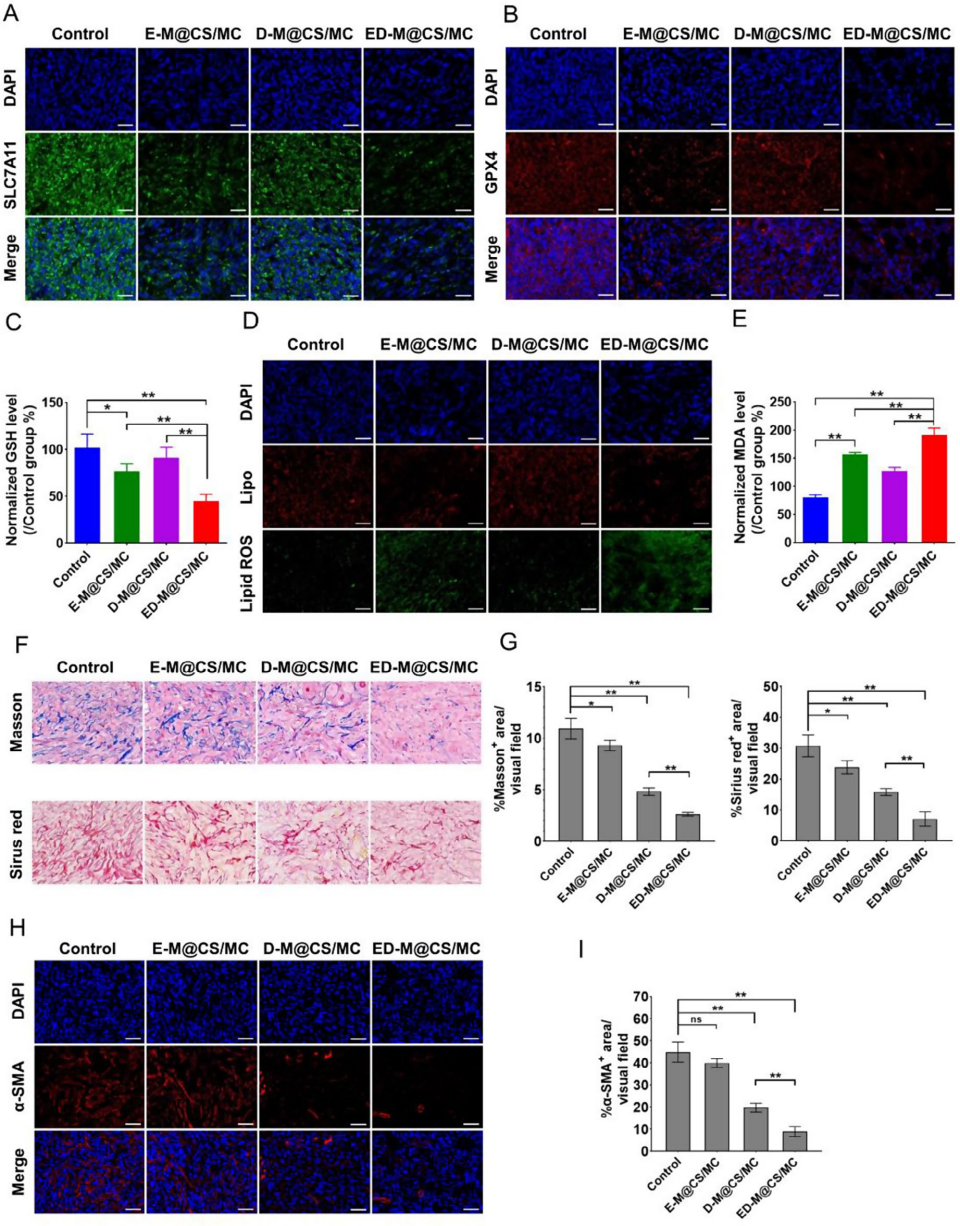
Ferroptosis activity and tumor stroma reduction in Panc02-bearing xenograft mice after different treatments. (**A**) Representative images of SLC7A11 expression by immunofluorescence staining. Scale bar: 50 μm. (**B**) Representative images of GPX4 expression by immunofluorescence staining. Scale bar: 50 μm. (**C**) The GSH level after different treatments (*n* = 5, *P < 0.05, **P < 0.01). (**D**) Representative images of C11-BODIPY^581/591^ dye-staining assay, and C11-BODIPY^581/591^ exhibited green fluorescence (Lipid ROS) and red fluorescence (Lipo) emission, respectively. Scale bar: 50 μm. (**E**) The MDA level after different treatments (*n* = 5, *P < 0.05, **P < 0.01). (**F**) Immunohistochemical staining of Massons trichrome and Sirius red. Scale bar: 50 μm. (**G**) Semi-quantitative analysis of Massons trichrome and Sirius red staining by ImageJ (*n* = 3, Mean ± SD, **P < 0.01, *P < 0.05). (**H**) Immunofluorescence staining of α-SMA. Scale bar: 50 μm. (**I**) Average fluorescence of α-SMA analyzed by ImageJ (*n* = 3, Mean ± SD, **P < 0.01, *P < 0.05, ns, not significant)

As a consequence of intracellular redox imbalance, ferroptosis initiates a cascade response involving diminished synthesis of GSH, decreased reduction of phospholipid hydroperoxide (PLOOH), and potential inactivation of glutathione peroxidase 4 (GPX4) [47, 48]. To investigate whether erastin induced ferroptosis in tumor cells, lipid peroxidation (Lipid ROS) and the corresponding antioxidant markers, GPX4 and GSH were examined [49]. System Xc^-^ is an amino acid transporter protein that brings cystine into the cell and exports glutamate, which plays a pivotal role in the synthesis of intracellular GSH [50]. SLC7A11 is a subunit of system Xc^-^, and the downregulation of SLC7A11 may reduce the activity of GPX4, thus leading to the induction of ferroptosis. Immunofluorescent staining analysis of SLC7A11 and GPX4 revealed that E-M@CS/MC effectively down-regulated SLC7A11 and GPX4 expression compared with the control, and that was more significantly observed in the ED-M@CS/MC (Fig. 3A, B). The changes in GSH levels of tumor tissue also showed that E-M@CS/MC decreased the GSH level to 76% compared with the control and the D-M@CS/MC group (91%), while ED-M@CS/MC decreased the GSH level to 47% of the control group (Fig. 3C). The prominent feature of ferroptosis is excessive lipid accumulation, the Lipid ROS process will eventually produce malondialdehyde (MDA) [51]. By utilizing C11-BODIPY^581/591^, an oxidation-sensitive and Lipid ROS-specific fluorescent probe, it was observed that E-M@CS/MC would lead to a increase of lipid peroxide levels, and the ED-M@CS/MC has a larger increase in lipid peroxidation (Fig. 3D). By analyzing the MDA content of experimental groups, it was observed that E-M@CS/MC significantly enhanced the MDA level by about 2.6 times than control, and the MDA level rose up to 3.1 times in the ED-M@CS/MC group (Fig. 3E). It can be concluded that E-M@CS/MC and ED-M@CS/MC effectively impeded the antioxidant production in ferroptotic pathway; however, the stronger ferroptotic peroxidation effect in the ED-M@CS/MC group suggested that the stroma manipulation by defactinib would explain for more effective anti-tumor.

### Stroma reduction by ED-M@CS/MC hydrogels in xenograft PDAC model

A typical characteristic of PDAC is the high density of tumor stroma. Collagen, as the primary structural component of tumor stroma, is excessively produced in PDAC, which restricts drug perfusion and accumulation in tumor tissue [52]. Both the Masson trichrome staining and Sirius red staining revealed that the collagen fiber area in the ED-M@CS/MC was the lowest among the treatment groups (Fig. 3F, G). As we all know, collagen proteins are mainly produced and secreted by cancer-associated fibroblasts (CAF). The α-SMA expressed by the CAF were further stained by immunochemical methods. The data indicated that the control group had 5.1 times more intensity than ED-M@CS/MC, and 2.3 times more than the D-M@CS/MC, which suggested a decrease in CAF in the treatment groups (Fig. 3H, I). The phosphorylation FAK level was also tested. There was no difference in FAK expression among all groups, and ED-M@CS/MC led to the lowest levels of FAK phosphorylation (p-FAK) among the treatment groups (Fig. S10). The p-FAK inhibited by defactinib could explain the reduction of CAF and the content of collagen [53, 54].

### ED-M@CS/MC hydrogels induced ICD and infiltration of anti-tumoral immune cells to promote anti-tumor immunity

**Fig. 4:**
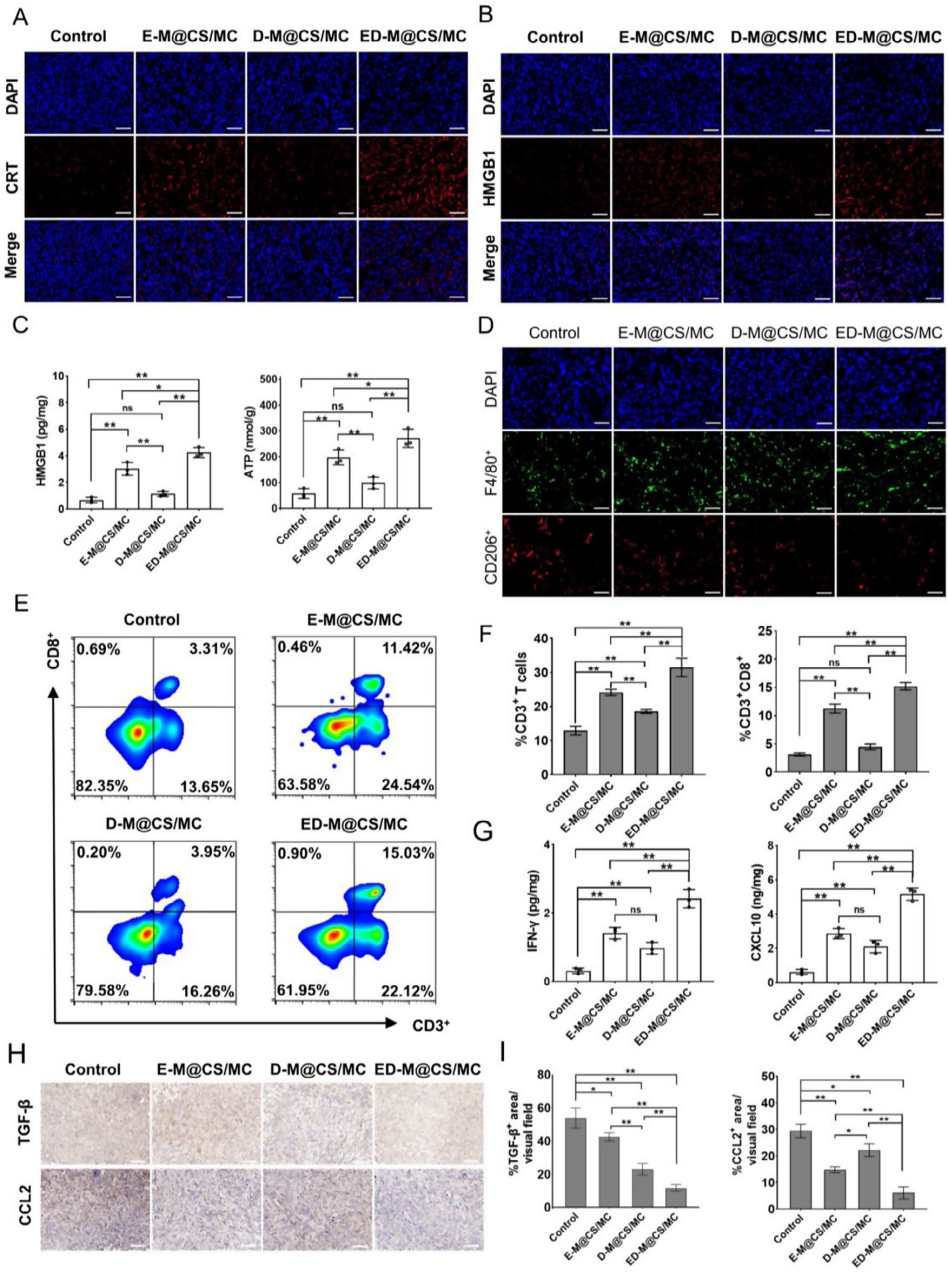
Anti-tumor immunity promotion of ED-M@CS/MC by ICD induction and CD3^+^CD8^+^ T cells infiltration. (**A**) Representative immunofluorescence staining of CRT. Scale bar: 50 μm. (**B**) Representative immunofluorescence staining of HMGB1. Scale bar: 50 μm. (**C**) ELISA analysis of HMGB1 and ATP levels (*n* = 3, Mean ± SD, *P < 0.05, **P < 0.01). (**D**) Immunofluorescence staining of M2 (F4/80^+^CD206^+^) macrophagocytes. Scale bar: 50 μm. (**E**) Representative flowcharts of CD3^+^CD4^+^ T cells in the tumor tissue. (**F**) FCM analysis of CD3^+^CD4^+^ T cells in the tumor tissue (*n* = 3, Mean ± SD, *P < 0.05, **P < 0.01). (**G**) ELISA analysis of IFN-γ and CXCL10 levels (*n* = 3, Mean ± SD, *P < 0.05, **P < 0.01). (**H**) Representative immunohistochemical staining of TGF-β and CCL2. Scale bar: 50 μm. (**I**) Quantification of immunohistochemical staining of TGF-β and CCL2. (*n* = 3, Mean ± SD, *P < 0.05, **P < 0.01)

Ferroptosis can stimulate pro-inflammatory cell death and induce immunogenic cell death (ICD) to release the immunogenic damage-associated molecular patterns (DAMPs) [9]. DAMPs including calreticulin (CRT) and high-mobility group box 1 (HMGB1) are highly conserved and play essential roles in the activation of T cell-mediated immune responses [55]. Through immunofluorescence staining experiments, the expression of HMGB1 and CRT was tested. The E-M@CS/MC group and the ED-M@CS/MC group displayed significantly higher levels of CRT expression and HMGB1 release compared to the other two groups (Fig. 4A and B, Fig. S11). Furthermore, the release of ATP and HMGB1 was detected by ELISA kits for different interventions. The levels of HMGB1 and ATP in E-M@CS/MC group and the ED-M@CS/MC group were significantly elevated compared to those in the control group and D-M@CS/MC (Fig. 4C). The above results demonstrated that the induction of ferroptosis by erastin could promote the ICD, thus triggering a cascade of events leading to the activation of anti-tumor T cells, which are essential for mounting an effective immune response against cancer cells.

As indispensable components of innate immunity, macrophages contribute significantly to the inflammatory response. Immunofluorescence staining results revealed a significant increase in macrophage population in groups E-M@CS/MC and D-M@CS/MC compared to the control group (Fig. 4D, Fig.S12); however, there was a notable reduction in M2 macrophages, which are known for their association with immunosuppression and tumor progression [56]. The increase in macrophages observed in group ED-M@CS/MC surpassed that of other groups significantly, while the number of M2 macrophages remained consistently low (Fig. 4D, Fig. S12). An increase in M1 macrophages can promote the development of a pro-inflammatory tumor microenvironment. Anti-tumor immunity based on tumor-specific T lymphocytes has emerged as an important role. Infiltrating CD8^+^ T cells are activated upon recognition of tumor neoantigens, leading to their specific effects against tumor cells. Therefore, an analysis of the infiltrating CD8^+^ T lymphocytes and immunosuppressive Treg cells were conducted in the tumor tissues. The T lymphocytes were analyzed using flow cytometry, revealing that both E-M@CS/MC and D-M@CS/MC treatments resulted in an increased population of T lymphocytes compared to the control group, with a significant increase observed in the ED-M@CS/MC group (Fig. 4F). Moreover, treatment with E-M@CS/MC and D-M@CS/MC led to an elevation in CD4^+^ T helper cells and CD8^+^ cytotoxic T cells level, which were found to be significantly lower in the control group. The combination of erastin and defactinib exhibited the most pronounced enhancement of CD4^+^ T helper cells and CD8^+^ cytotoxic T cells infiltration within the tumor microenvironment (Fig. 4E, Fig. S13). Furthermore, immunofluorescent staining of CD4^+^Foxp3^+^regulatory T cells showed that their proportion was lowest in the ED-M@CS/MC group (Fig. S14). However, it was observed that levels of CD4^+^ T helper cells and CD8^+^ cytotoxic T cells were slightly lower in D-M@CS/MC compared to E-M@CS/MC; nevertheless, macrophage levels were significantly higher in D-M@CS/MC than in E-M@CS/MC. In addition, E-M@CS/MC and D-M@CS/MC remarkedly promoted the levels of IFN-γ and CXCL10 compared with the control group, and ED-M@CS/MC group produced highest level of IFN-γ (Fig. 4G). Similarly, FAK-dependent TGF-β2 and CCL2 were greatly reduced in ED-M@CS/MC group (Fig. 4H and I). These findings underscored the pivotal role of E-M@CS/MC and D-M@CS/MC in modulating various cytokines, showing potential implications for immune response and cellular function.

The previous research findings indicate that D-M@CS/MC exhibits a more potent anti-tumor effect compared to group E-M@CS/MC, it suggests that apart from inhibiting stroma formation, defactinib may enhance its anti-tumor immune effect by promoting macrophage infiltration. These findings suggest that erastin could induced ICD to activate the innate immune response, and facilitating the infiltration of cytotoxic CD8^+^ T cells to exert a potent anti-tumor effect. When combined with these two drugs, erastin can penetrate deeper into the tumor due to stroma reduction. This not only enhances activation of the innate immune response but also promotes cytotoxic CD8^+^ T cells infiltration and macrophage, thus maximizing the anti-tumor immune response.

### Combination treatment of pancreatic cancer through ferroptosis and stromal modulation in orthophoric primary PDAC mice

#### The anti-tumor efficacy of ED-M@CS/MC hydrogels in orthophoric primary PDAC

**Fig. 5:**
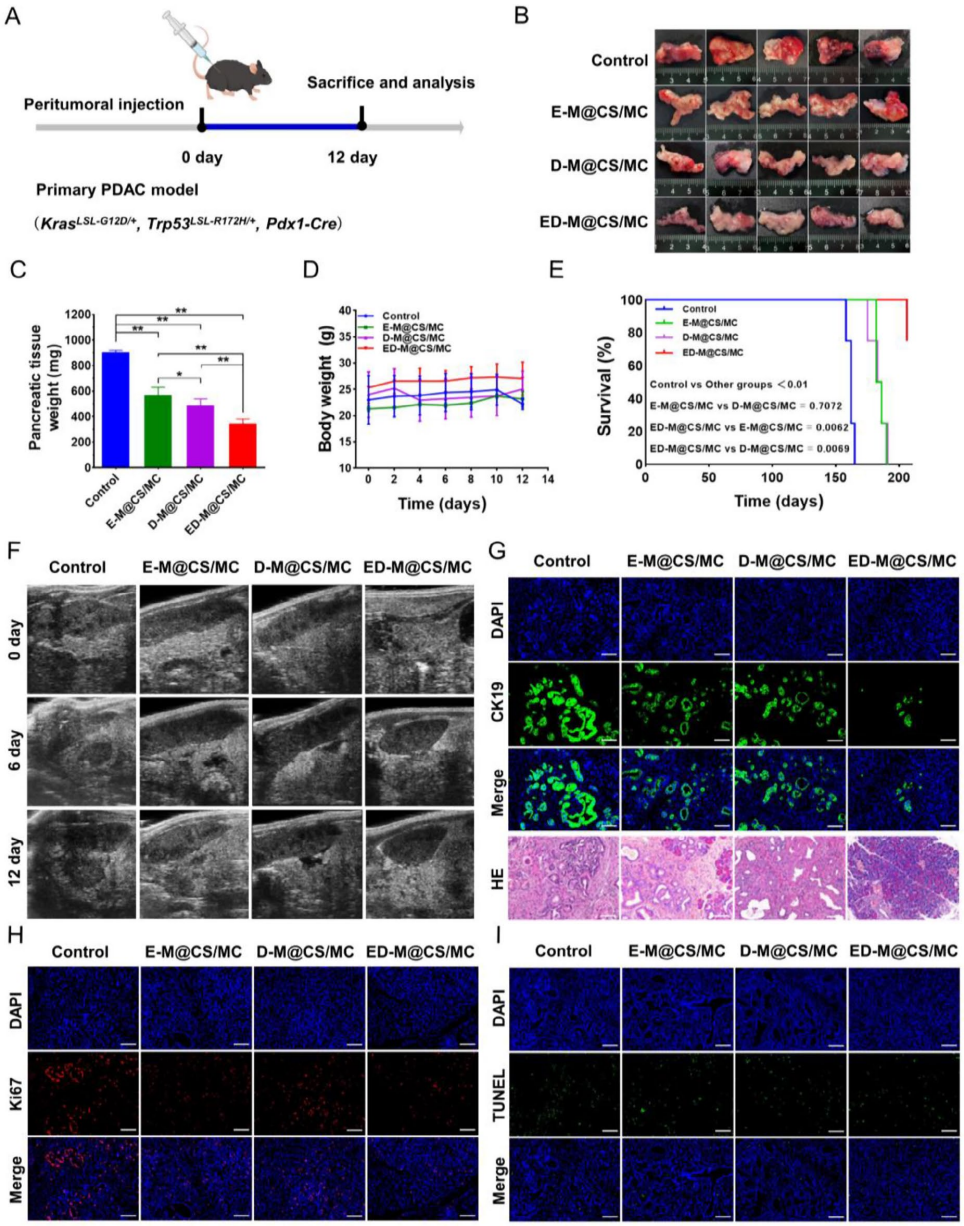
Anti-tumor efficacy in orthophoric primary PDAC mice. (**A**) Schedule for model establishment, drug treatment and tissue collection, and analysis. (**B**) Images of the pancreatic tissue. (**C**) Primary PDAC tissue weight (*n* = 5, Mean ± SD, **P < 0.01). (**D**) Body weight change curve after different treatments (*n* = 5). (**E**) Survival curves starting from the birth of KPC mice (*n* = 4). (**F**) Representative images of primary PDAC tissues after different treatments tested by ultrasound in B-mode. (**G**) Representative immunofluorescence images of CK19 and H&E staining of primary PDAC tissue. Scale bar: 100 μm. (**H**) Representative images of Ki67 expression tested by immunofluorescence staining. Scale bar: 100 μm. (**I**) Representative images of TUNEL staining. Scale bar: 100 μm

PDAC is distinguished from other tumor types by its remarkable stroma, which can account for up to 80% of the total tumor mass [57]. To further accurately mimic human disease with comparable oncogene expression, tumor growth characteristics, and desmoplastic stroma formation, an orthophoric primary PDAC model harboring with *LSL-Kras*^*G12D*^*(KI/+), LSL-Trp53*^*R172H*^*(KI/+), Pdx1-Cre (TG/+)* mutations [58] was employed to investigate the anti-tumor effect of ED-M@CS/MC. After a single intratumoral injection of D-M@CS/MC, E-M@CS/MC and ED-M@CS/MC according to the provided schedule (Fig. 5A), orthophoric primary PDAC tissues were collected for weight measurement, photography, and analysis (Fig. 5B). The average weights of orthophoric primary PDAC tissues after being treated with D-M@CS/MC and E-M@CS/MC were 494 ± 68 mg and 572 ± 68 mg, respectively. Remarkably, the combination therapy using ED-M@CS/MC exhibited an average weight of only 343 ± 21 mg, which closely resembled the weight of normal pancreas tissue of mice (Fig. 5C). The body weights of the mice were recorded during the treatment, and non-significant changes were found between the treatment groups and control group (Fig. 5D). The survival time starting from the birth of KPC mice are shown in Fig. 5E. The median survival time was 162 days for the control group, 184 days for the E-M@CS/MC group, 185 days for the D-M@CS/MC group, and only one mouse in the ED-M@CS/MC group died on the 206th day. All treated groups had significantly longer survival than that of the control group, and the ED-M@CS/MC group had significantly longer survival than E-M@CS/MC and D-M@CS/MC group.

The ultrasound imaging also revealed significant variations in pancreatic tissues after different treatments. In the ED-M@CS/MC group, there was a substantial reduction of orthophoric primary PDAC in the pancreas area, whereas the control group exhibited nearly complete occupation by the orthophoric primary PDAC. Both E-M@CS/MC and D-M@CS/MC groups showed a smaller extent of orthophoric primary PDAC tissues (Fig. 5F). Cytokeratin-19 (CK19) is an epithelial marker renowned for its ability to stain ducts. Duct metaplasia and duct function appearance of new duct/tubular structures is an important feature of PDAC [59]. Staining results showed a markedly reduced expression of CK19 after being treated with ED-M@CS/MC (Fig. 5G), which also exhibited the most pronounced inhibitory effect on pancreatic tumor as confirmed by H&E staining (Fig. 5G) and Ki67 staining (Fig. 5H). The TUNEL assays revealed that low tumor cell apoptosis was observed and no differences in induced specific apoptosis among all treatment groups (Fig. 5I). The major organs including the heart, liver, spleen, lung, and kidney were collected for H&E staining. No apparent histological damage was observed in the major organs (Fig. S15). The peripheral blood was also collected for serum biochemical analysis. The concentrations of ALT, AST, BUN, and CREA were still within the normal physiological range (Fig. S16).

### Stroma reduction by ED-M@CS/MC hydrogels in orthotopic primary PDAC model

**Fig. 6:**
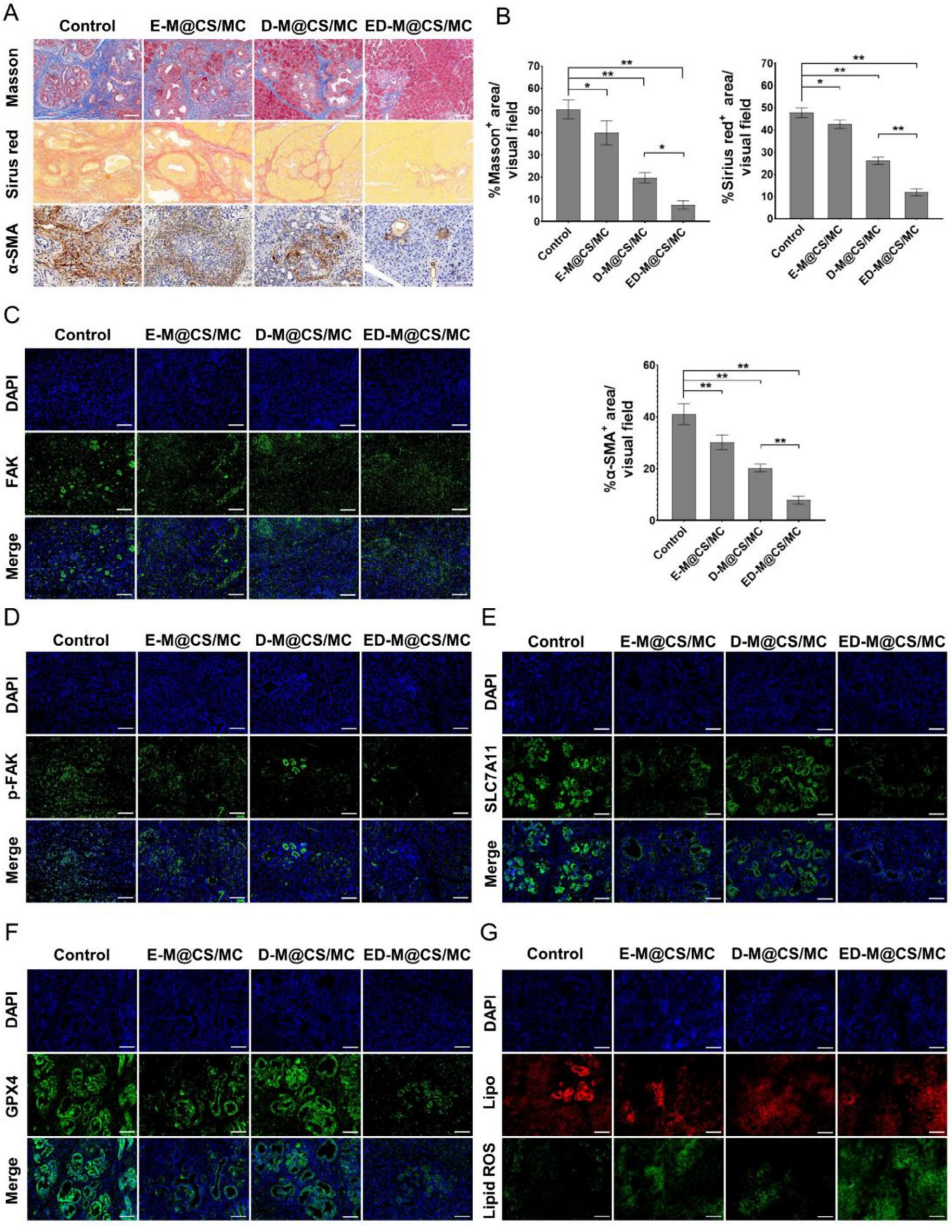
Tumor stroma reduction and ferroptosis activity in orthophoric primary PDAC mice after different treatments. (**A**) Immunohistochemical staining of Masson trichrome, Sirius red and α-SMA. Scale bar: 100 μm. (**B**) Semi-quantitative analysis of Masson staining, Sirius red and α-SMA by ImageJ (*n* = 3, Mean ± SD, *P < 0.05, **P < 0.01). (**C**) Immunohistochemical staining of FAK. Scale bar: 100 μm. (**D**) Immunohistochemical staining of p-FAK. Scale bar: 100 μm. (**E**) Representative images of SLC7A11 expression tested by immunofluorescence staining. Scale bar: 100 μm. (**F**) Representative images of GPX4 expression. Scale bar: 100 μm. (**G**) Representative images of C11-BODIPY^581/591^ dye staining, C11-BODIPY^581/591^ exhibit green fluorescence (Lipid ROS) and red fluorescence (Lipo) emission, respectively. Scale bar: 100 μm

To investigate the impact of ED-M@CS/MC on the stromal component, Masson trichrome staining and Sirius red staining were performed. The collagen of orthophoric primary PDAC in the ED-M@CS/MC was the lowest among the treatment groups (Fig. 6A, B). The production of collagen is attributed to CAF, which exhibits enhanced proliferation, migration, and secretion of fibroblast factors, along with higher levels of α-SMA and collagen, leading to connective tissue hyperplasia [60]. Histological analysis also revealed the largest reduction of α-SMA expression when treated with ED-M@CS/MC (Fig. 6A, B). Recent studies have confirmed that CAF can mediate collagen crosslinking and modify the extracellular matrix via p-FAK signaling then promote the cancer progression [61]. The phosphorylation of FAK is crucial for the abnormal accumulation of tumor stroma, hyperplasia of connective tissue, and tumor growth. Inhibiting the phosphorylation of FAK by defactinib can regulate the stromal density [62, 63]. The staining results revealed that ED-M@CS/MC could effectively suppress the phosphorylation of FAK (Fig. 6C, D), thereby modulating tumor stroma formation.

### Ferroptosis-triggering anti-tumor effect of ED-M@CS/MC hydrogels in orthotopic primary PDAC model

To further investigate the pivotal role of ferroptosis by erastin in orthophoric primary PDAC mice, the expression levels of SLC7A11, GPX4, and lipid peroxides were examined. The findings revealed a significant down-regulation of GPX4 expression after ED-M@CS/MC treatment, accompanied by successful inhibition of SLC7A11 activity (Fig. 6E, F). This discovery holds immense significance for the role of ferroptosis in pancreatic cancer initiation and progression. Furthermore, fluorescent lipid peroxidation sensor BODIPY^581/591^ C11 exhibited higher oxidation levels after treated with ED-M@CS/MC and E-M@CS/MC (Fig. 6G). This finding further confirmed that ED-M@CS/MC exhibited significant advantages in promoting the efficacy of ferroptosis in tumor tissue because reducing the extracellular matrix may help the drug penetration into the tumor.

### Improvement of tumor-specific immune microenvironment by ED-M@CS/MC hydrogels in orthotopic primary PDAC model

**Fig. 7:**
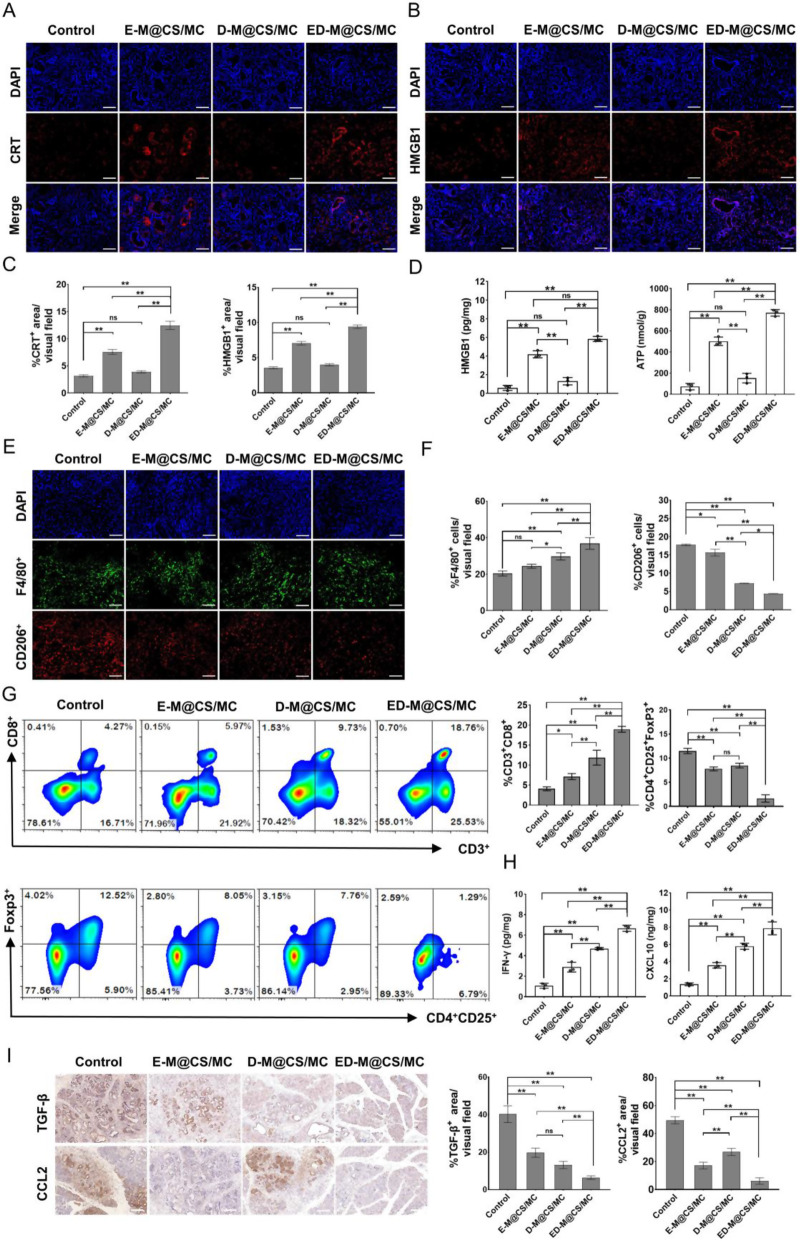
Improvement of the tumor-specific immune microenvironment in orthotopic primary PDAC mice after different treatments. (**A**) Representative immunofluorescence staining images of CRT. Scale bar: 100 μm. (**B**) Immunofluorescence staining images of HMGB1. Scale bar: 100 μm. (**C**) Positive area of CRT and HMGB1 in the field of vision analyzed by ImageJ (*n* = 3, Mean ± SD, *P < 0.05). (**D**) ELISA analysis of HMGB1 and ATP levels (*n* = 3, Mean ± SD, *P < 0.05, **P < 0.01). (**E**) Immunofluorescence staining images of M2 (F4/80^+^CD206^+^) macrophagocytes. Scale bar: 100 μm. (**F**) Positive area of macrophagocytes and M2 (F4/80^+^CD206^+^) macrophagocytes in field of vision analyzed by ImageJ (*n* = 3, Mean ± SD, *P < 0.05). Scale bar: 100 μm. (**G**) Representative flowcharts of CD3^+^CD4^+^ T cells and regulatory T cells (CD4^+^CD25^+^Foxp3^+^) and FCM analysis of CD3^+^CD4^+^ T cells and regulatory T cells (CD4^+^CD25^+^Foxp3^+^) in the tumor tissue (*n* = 3, Mean ± SD, *P < 0.05, **P < 0.01). (**H**) ELISA analysis of IFN-γ and CXCL10 levels (*n* = 3, Mean ± SD, *P < 0.05, **P < 0.01). (**I**) Representative immunohistochemical staining of TGF-β and CCL2 and the quantification of immunohistochemical staining of TGF-β and CCL2 (Scale bar: 100 μm, *n* = 3, Mean ± SD, *P < 0.05, **P < 0.01)

The primary PDAC model provided a comparable representation of human tumor microenvironment dynamics alongside a depiction of immune responses in the synergism treatment of PDAC. PDAC is a typical cold tumor featuring a paucity of immune cell infiltration and immunosuppressive microenvironment [64]. In xenograft mice, the combination of erastin and defactinib has been demonstrated to enhance anti-tumor immunity by inducing ICD and reducing the tumor stroma. As we all know, the orthotopic primary PDAC mice have demonstrated clinical relevance to the human tumor microenvironment of patients [65]. The immune activation property of ED-M@CS/MC was further explored in orthotopic primary PDAC mice. As shown in the immunofluorescence staining of the orthotopic primary PDAC tissue sections, the elevated levels of CRT and released HMGB1 were found both in E-M@CS/MC and ED-M@CS/MC which contain erastin (Fig. 7A-C), thereby providing evidence that ferroptosis can effectively induce ICD. The ELISA results also showed a notable increase in the concentrations of HMGB1 and ATP in E-M@CS/MC and ED-M@CS/MC group in comparison to the other two groups (Fig. 7D). It could be attributed to the activation of the ferroptosis to effectively induce ICD in the orthotopic primary PDAC model.

To further understand the infiltration of immune cells in the tumor microenvironment, the distribution and number of tumors infiltrating tumor-associated macrophages (TAMs) were detected through immunofluorescence staining. Increasing evidence suggests that manipulating the phenotype of tumor-associated macrophages (TAMs) is crucial in triggering efficient anti-tumor immune responses. Type 2 macrophages (M2) in tumor is one of the essential components in forming the immunosuppressive microenvironment, and their population is closely correlated to the poor prognosis of PDAC [66]. After experimental analysis, an increase in the number of macrophages and a decrease in the number of M2 (F4/80^+^CD206^+^) macrophages was observed. Among them, the highest count of macrophages and the lowest count of M2 (F4/80^+^CD206^+^) macrophages were observed in the group of ED-M@CS/MC (Fig. 7E and F, Fig. S17), which could be caused by inhibiting FAK phosphorylation, which may promote the repolarization of M2 to M1 macrophages, leading to a reeducation of the microenvironment towards a pro-inflammatory state. The proposed mechanism underlying FAK-mediated TAM repolarization involves directly inhibiting FAK signaling and subsequent regulation of the PI3K/Akt, STAT3, and NF-κB pathways [67–70].

Furthermore, T lymphocytes in orthotopic primary PDAC tissues were also tested by flow cytometry (Fig. 7G). The result showed that an increased CD3^+^CD4^+^ T cell infiltration after treated with E-M@CS/MC (14.00%) and D-M@CS/MC (13.56%) was found compared to the control group (11.08%), and ED-M@CS/MC the most significant increase of CD3^+^CD4^+^ T cell infiltration (22.58%) (Fig.S19). The result also showed that a higher CD3^+^CD8^+^ T cell infiltration after treated with E-M@CS/MC (5.97%) and D-M@CS/MC (9.73%) was found compared to the control group (4.27%), and ED-M@CS/MC the most significant increase of CD3^+^CD8^+^ T cell infiltration (18.76%) (Fig. 7G). As for the regulation of T cells, the percentage of Treg cells was lower in the E-M@CS/MC group (8.05%) and D-M@CS/MC group (7.76%) compared to the control group (12.52%). Although there was no statistical difference between the two groups, reduced Treg cells were observed in the combined ED-M@CS/MC group (1.29%) (Fig. 7G, Fig. S20). ELISA analysis of cytokines revealed that E-M@CS/MC and D-M@CS/MC significantly and consistently increased the levels of IFN-γ and CXCL10 compared to the control group. The ED-M@CS/MC group demonstrated an exceptionally prominent elevation of IFN-γ levels (Fig. 7H), which was accompanied by a significant reduction in the levels of TGF-β2 and CCL2 in the same group compared to other groups (Fig. 7I). These results suggested that E-M@CS/MC and D-M@CS/MC could play crucial roles in modulating the expression of IFN-γ, TGF-β2, and CCL2, and ED-M@CS/MC exhibited an exceptional ability to stimulate and enhance anti-tumor immune responses to inhibit the tumor growth. Taking together the findings of significant infiltration of anti-tumoral lymphocytes including cytotoxic T cells and type 1 macrophages, it demonstrated that the ED-M@CS/MC hydrogels not only effectively inhibited the tumor growth, but also aroused anti-tumor immune responses, which attributed to the combination ability to reduce tumor stroma and induce the ferroptosis in orthotopic primary PDAC.

## Conclusion

PDAC is a most resistant malignant tumor to chemotherapy and immunotherapy, which is closely related to the particularity of pancreatic tumor tissue and immune microenvironment. In order to improve the therapeutic performance, an effective combination strategy was proposed by using an in situ injectable ED-M@CS/MC hydrogels loaded with erastin and defactinib to take the tumoracidal effect, stromal manipulation, and anti-tumor immunity into consideration. After prescription optimization, the ED-M@CS/MC hydrogel system achieved physical crosslinking with no chemical modification that alters the approved excipients and allowed the retention in vivo and the release of the drugs for up to 12 days. After only a single intratumoral injection, erastin and defactinib can be sustained release and play a synergistic therapeutic role. Erastin aggravated the hyper oxidation of tumor cells, induced the ferroptosis to suppress the proliferative signals, and triggered innate and adaptive immune responses by releasing immunogenic molecules including CRT and HMGB1. Defactinib inhibited FAK phosphorylation, ameliorated pancreatic morphology by modulating the tumor stroma, and also facilitated the immune infiltration. Combining erastin and defactinib significantly remodeled the pancreatic morphology towards the normal, and shifted the suppressive immune microenvironment into a “hot” state by promoting the intratumoral infiltration of CD8^+^ T cells and decreasing Treg lymphocytes and intratumoral type II macrophages, which plays a crucial role in arousing anti-tumor activity. The in situ combination strategy encourages the potential clinical translation upon unresectable pancreatic tumor and provided a new promising approach for PDAC chemotherapy.

### Electronic supplementary material

Below is the link to the electronic supplementary material.

Supplementary Material 1

## Data Availability

Data is provided within the manuscript or supplementary information files.
