# Supplementary material for: Injectable hybrid hydrogels enable enhanced combination chemotherapy and roused anti-tumor immunity in the synergistic treatment of pancreatic ductal adenocarcinoma
Source: J Nanobiotechnology. 2024 Jun 20;22:353. doi: 10.1186/s12951-024-02646-7 (PMC11191229; doi:10.1186/s12951-024-02646-7)
Supplement: Supplementary file 1 — Supplementary Material 1 [file 12951_2024_2646_MOESM1_ESM.docx]

Supporting Information

**Injectable hybrid hydrogels enable enhanced combination chemotherapy and roused anti-tumor immunity in the synergistic treatment of pancreatic ductal adenocarcinoma**

*Hao Zhou^a^, Wei Wang^a^, Zedong Cai^a^, Zhou-Yan Jia^a^, Yu-Yao Li^a^, Wei He^b, c,*^, Chen Li^b^ and Bang-Le Zhang^a, b, *^*

^a^ Department of Pharmaceutics, School of Pharmacy, Fourth Military Medical University, Xi’an, 710032, China; ^b^ Key Laboratory of Pharmacology of the State Administration of Traditional Chinese Medicine, Fourth Military Medical University, Xi’an, 710032, China; ^c^ Department of Chemistry, School of Pharmacy, Fourth Military Medical University, Xi’an, 710032, China

*Corresponding authors: blezhang@fmmu.edu.cn (BL Zhang); weihechem@fmmu.edu.cn (W He).


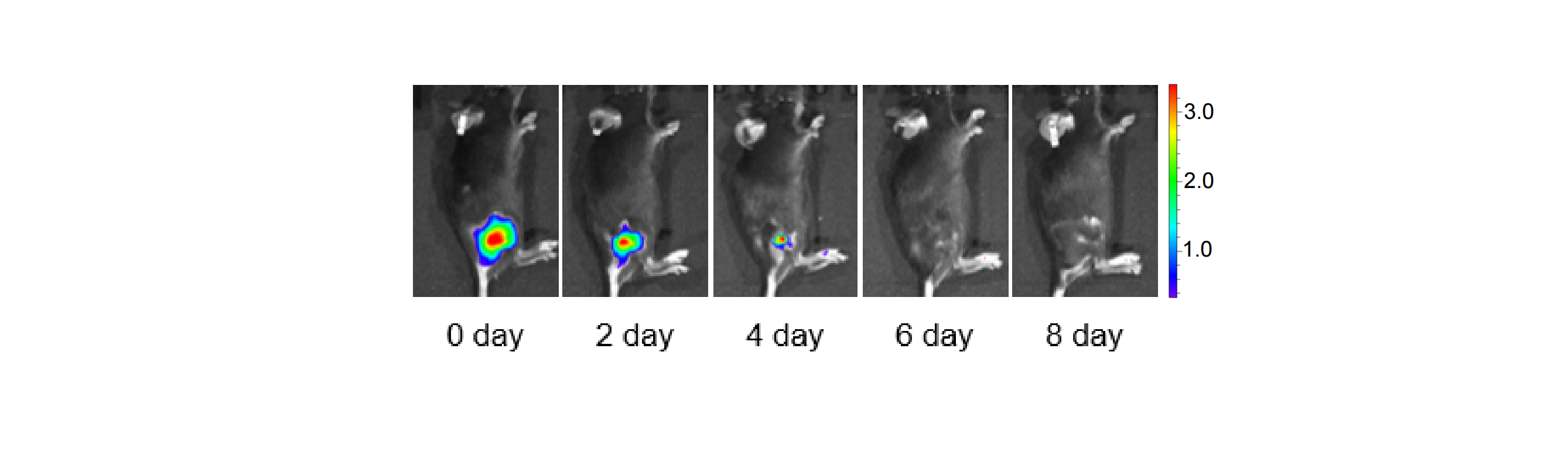


**Figure S1.** Fluorescence IVIS imaging depicting the in vivo degradation of Cy5.5@CS/β-GP


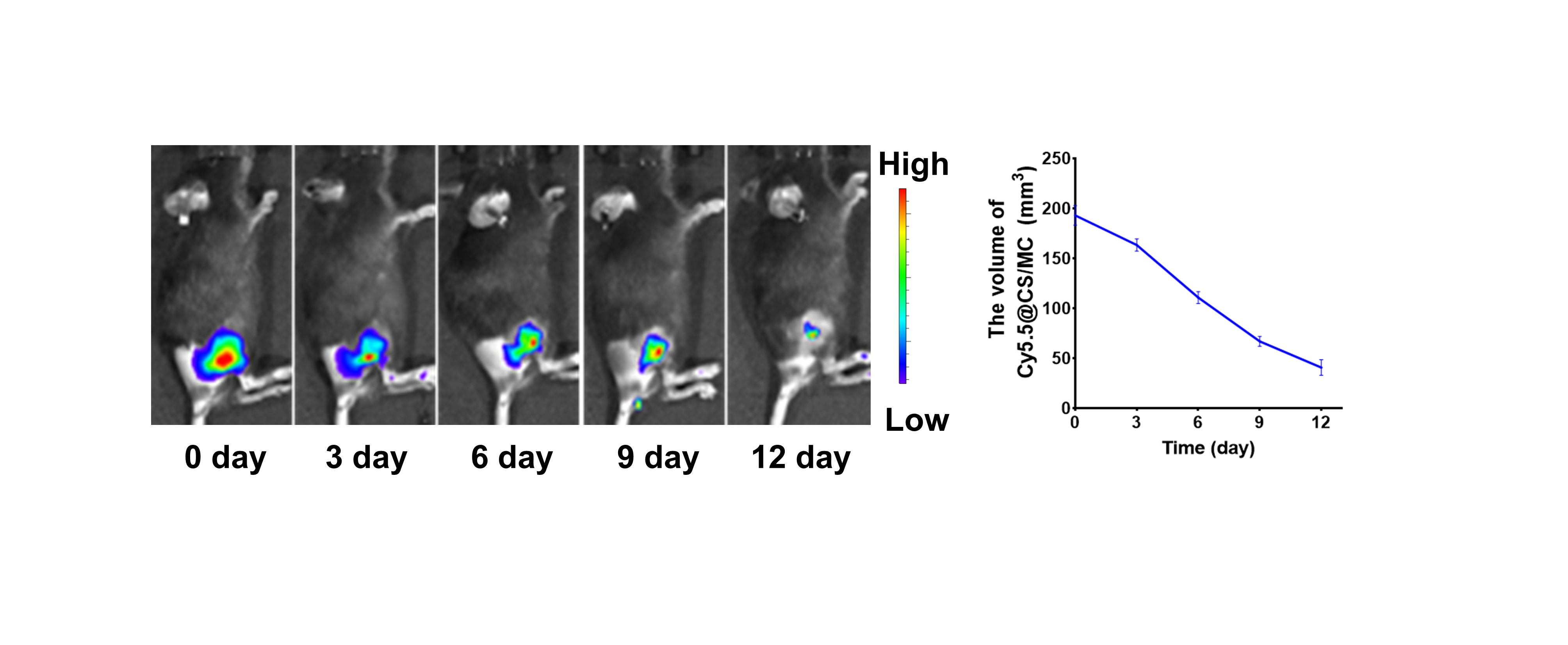


**FigureS2.** Fluorescence IVIS imaging of Cy5.5@CS/MC hydrogels in vivo and the change of hydrogel volume in vivo (n = 3)


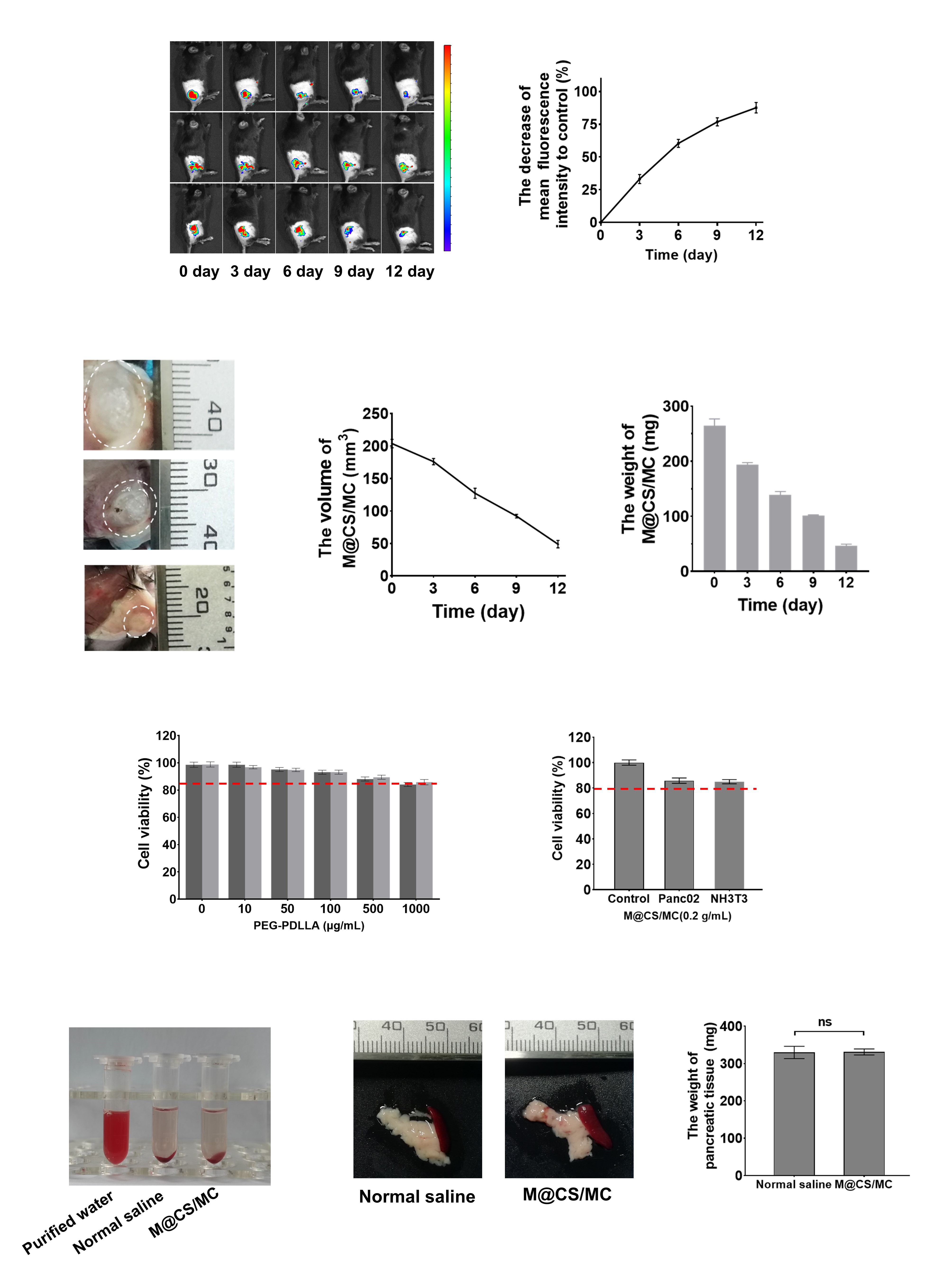


**Figure S3.** The cumulative release profiles of the Cy3-M@CS/MC hydrogels in vivo (n = 3)


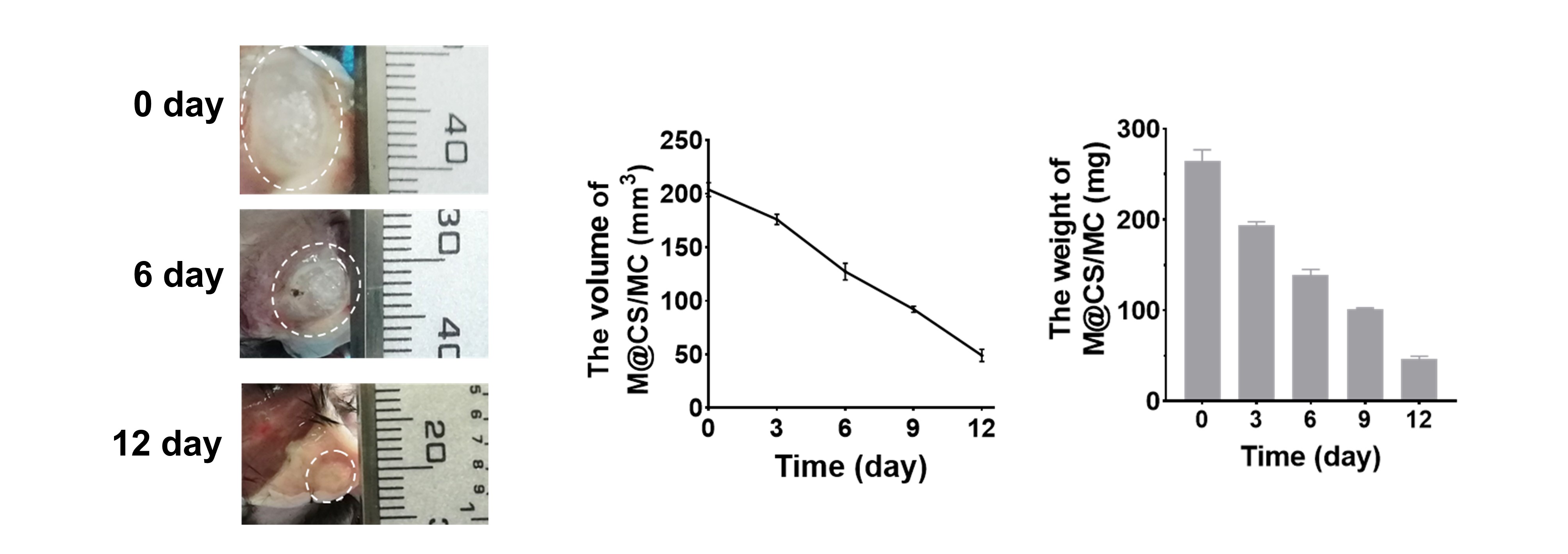


**Figure S4.** The degradation of M@CS/MC hydrogels in vivo by testing the change of the volume and weight (n = 3)

**
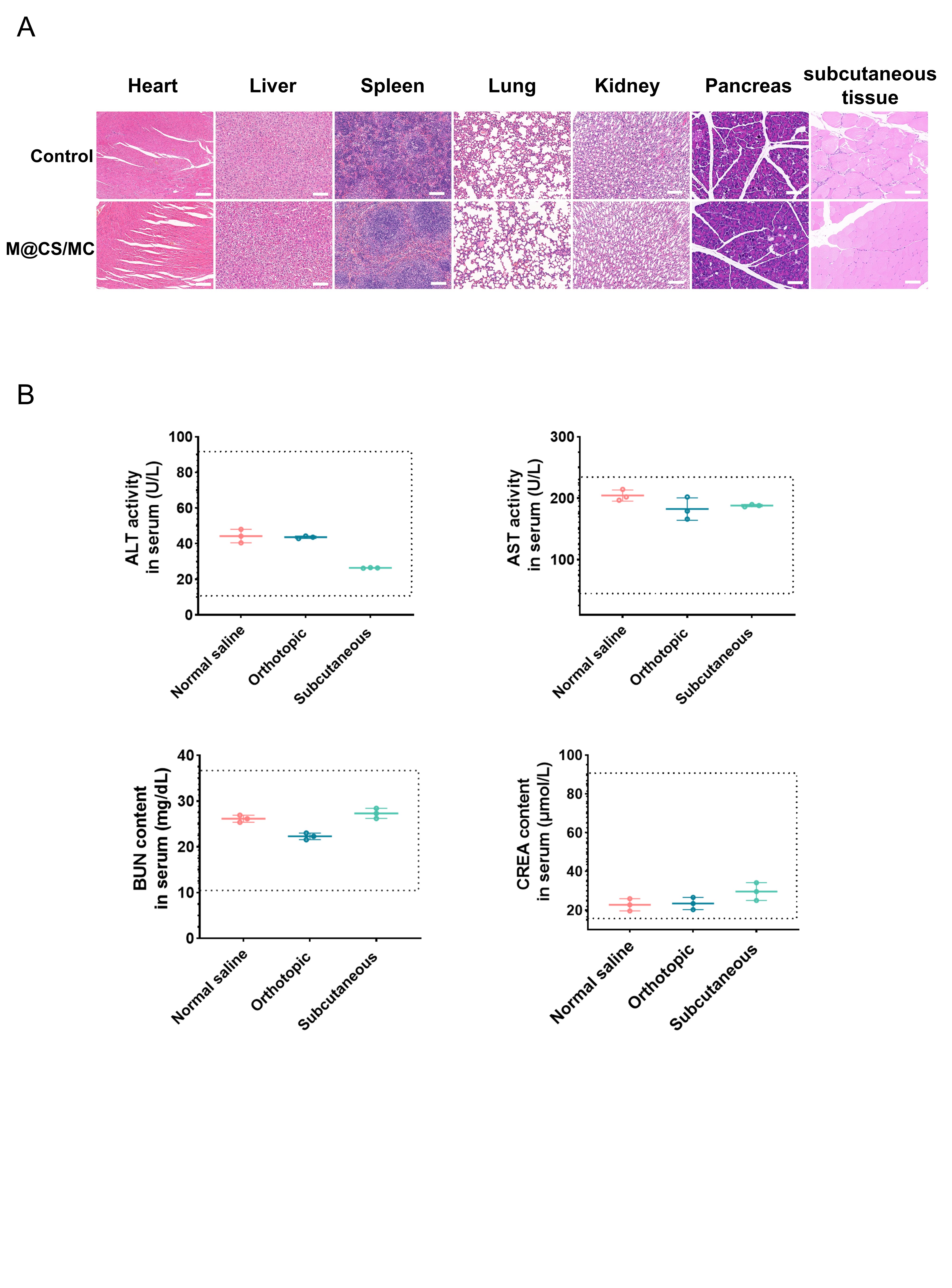
**

**Figure S5.** H&E staining analysis of major organs from the C57BL/6 mice after treated with M@CS/MC. Scale bar: 50 μm.


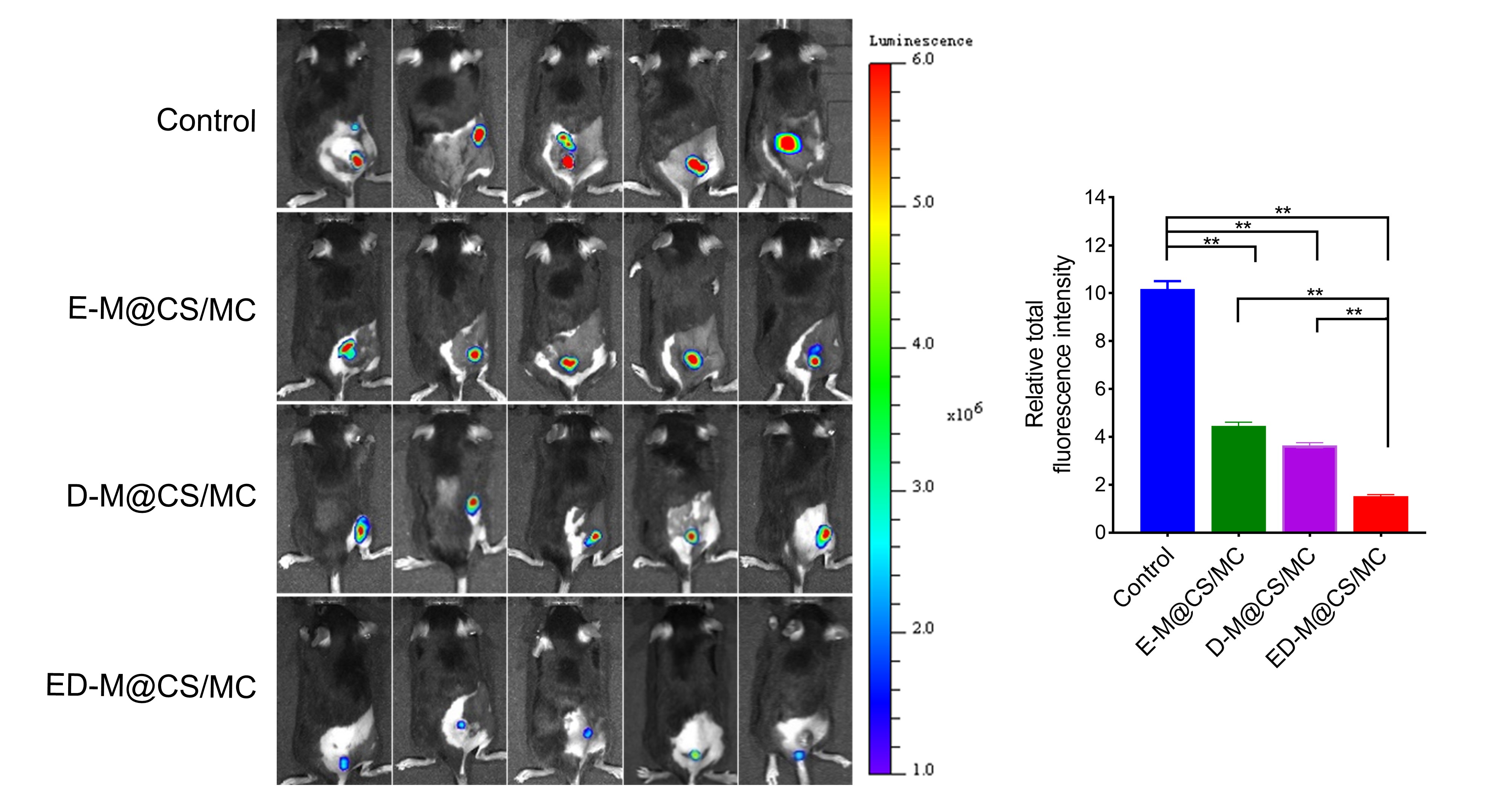


**Figure S6.** Tumor bioluminescence images and bioluminescence intensity after different treatments (n = 5, Mean ± SD, ** *P* < 0.01)


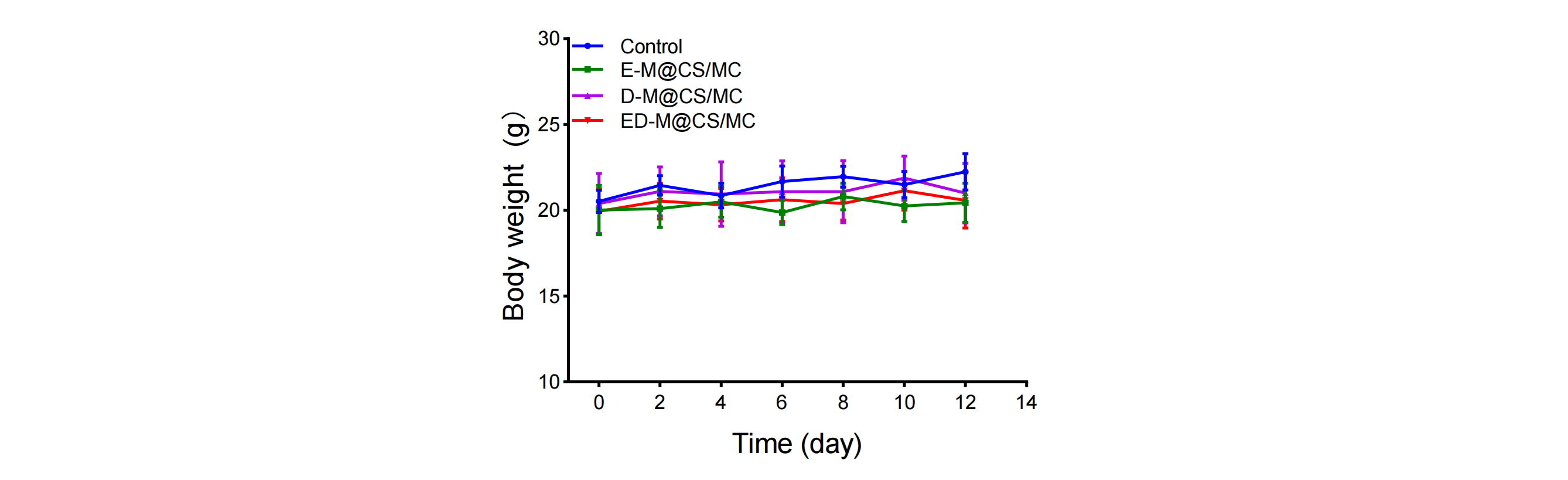


**Figure S7.** Body weight change curve after different treatments (n = 5)

**
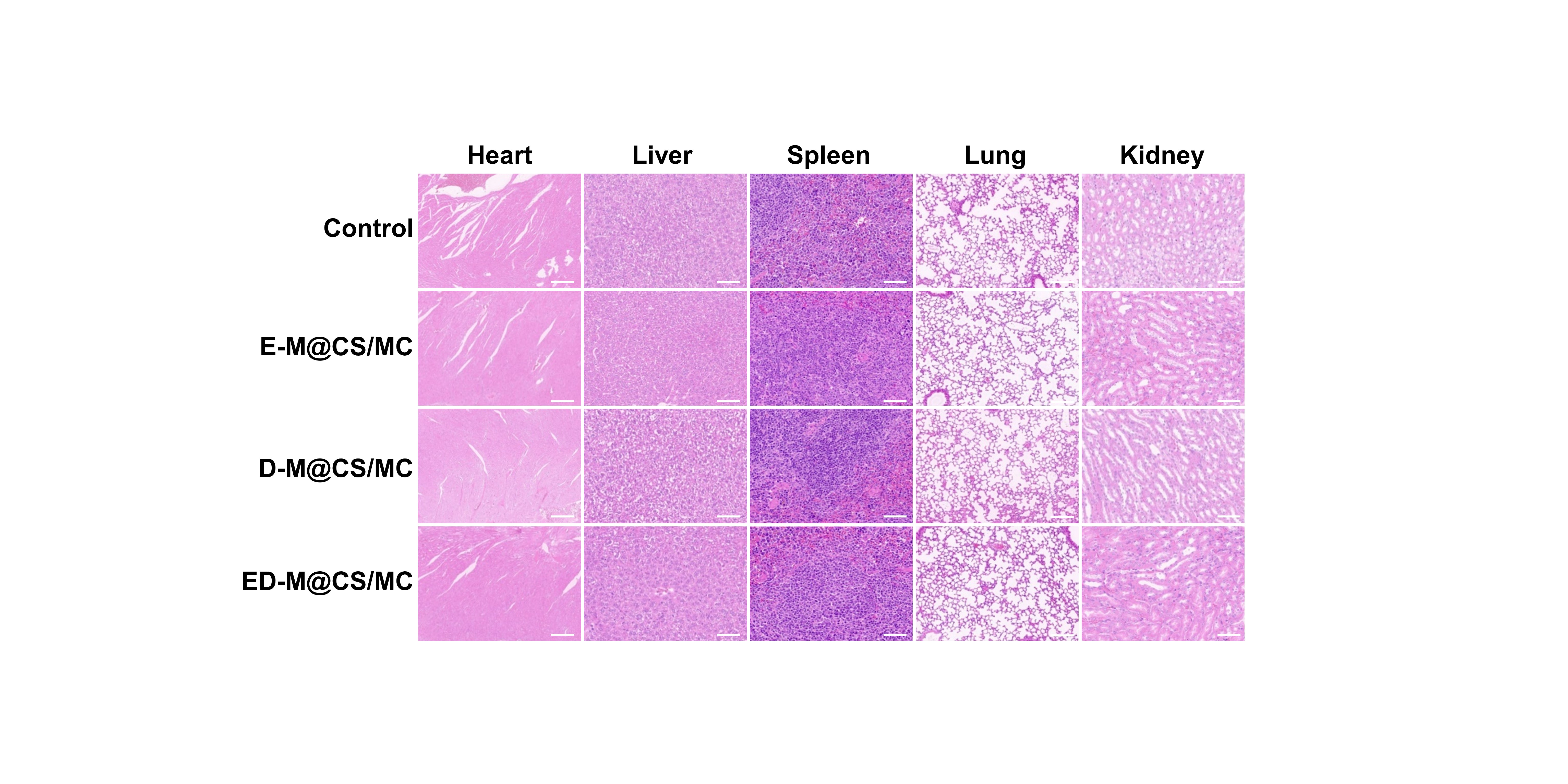
**

**Figure S8.** H&E staining analysis of the major organs after different treatments. Scale bar: 50 μm.


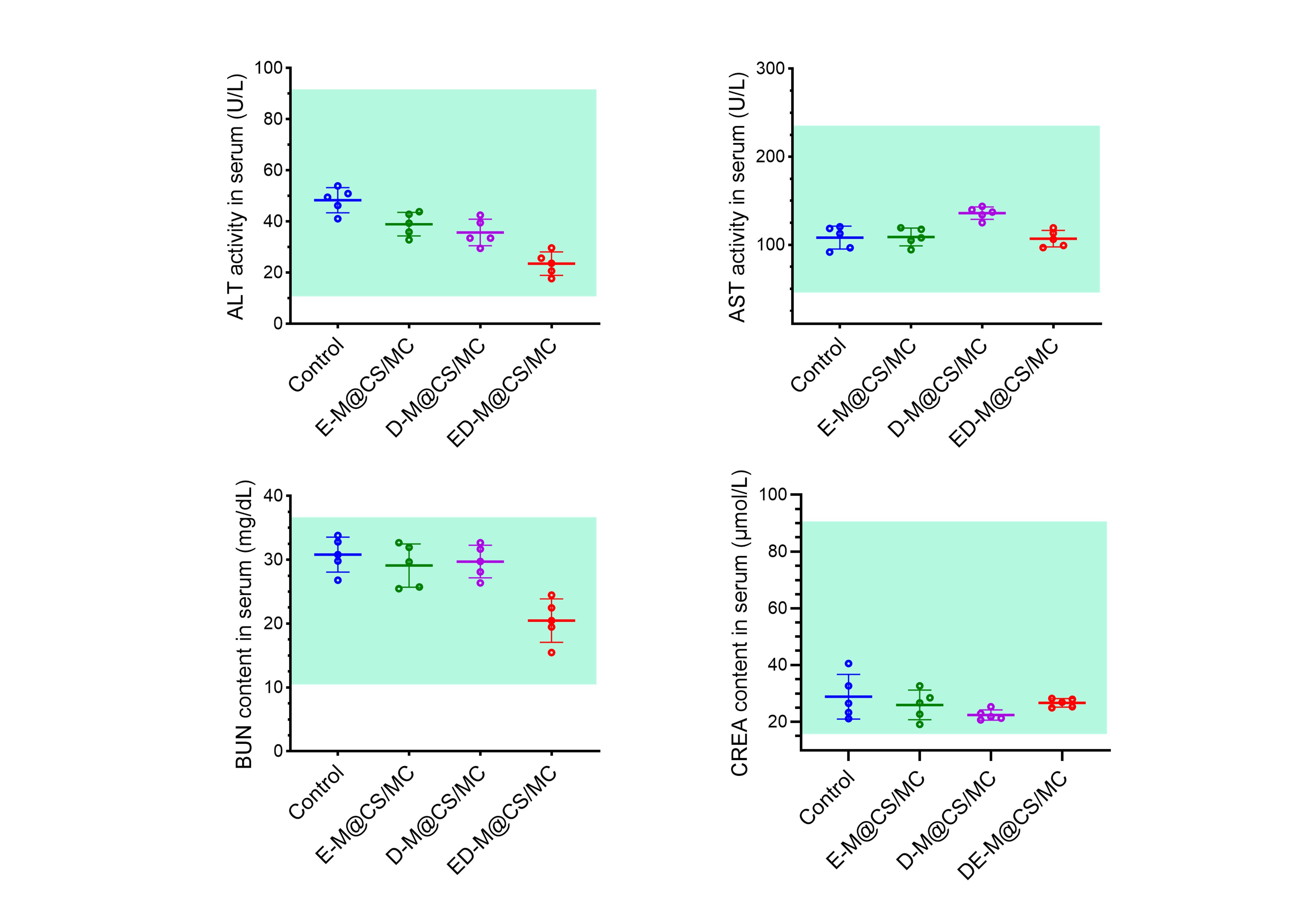


**Figure S9.** The serum concentrations of ALT, AST, BUN and CREA after different treatments in the xenograft mice (n = 5, Mean ± SD).


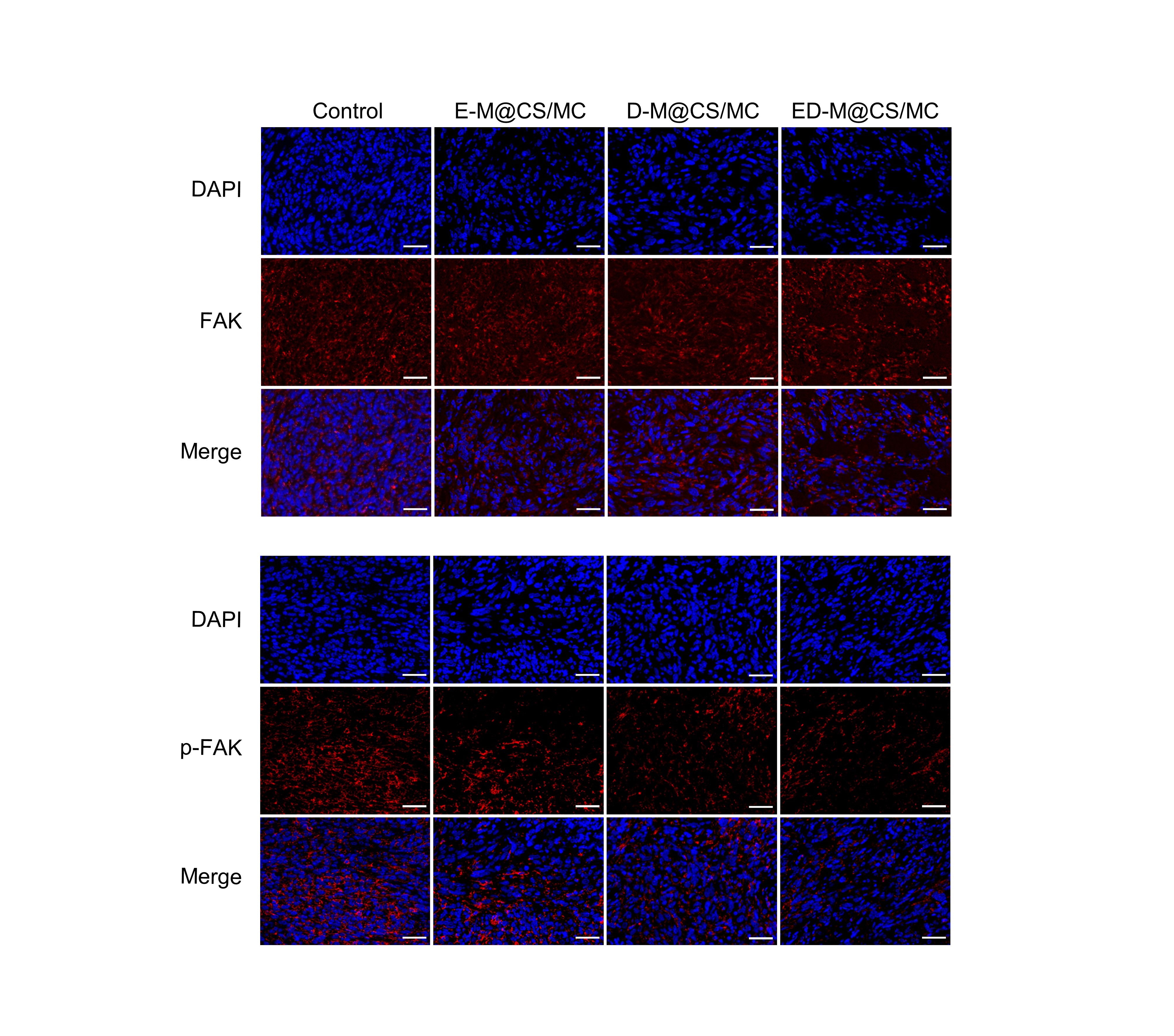


**Figure S10.** Representative immunofluorescence staining images of FAK and p-FAK in tumor tissue after different treatments. Scale bar: 50 μm.


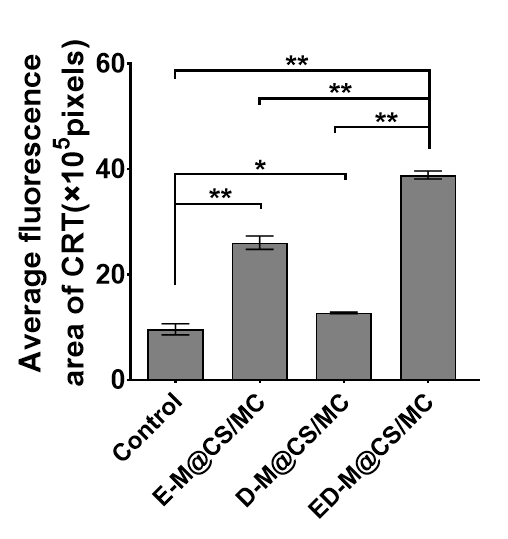

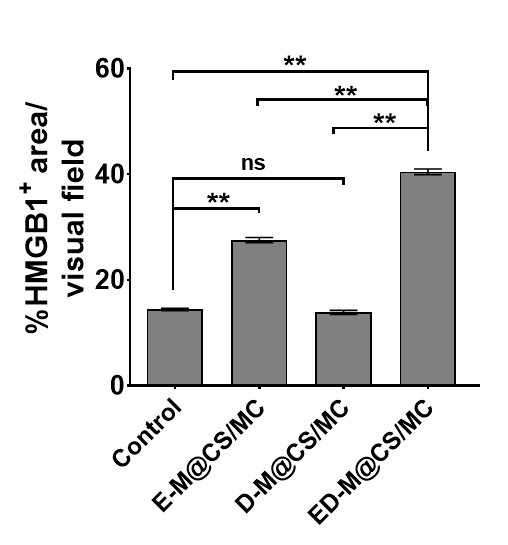


**Figure S11.** CRT and HMGB1 in tumor tissue after different treatments analyzed by ImageJ (n = 3, Mean ± SD, **P*< 0.05)


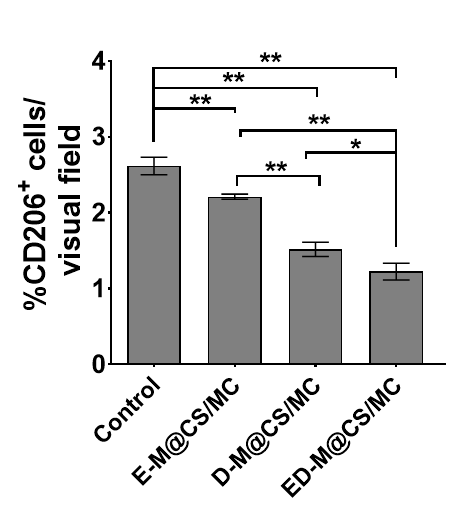

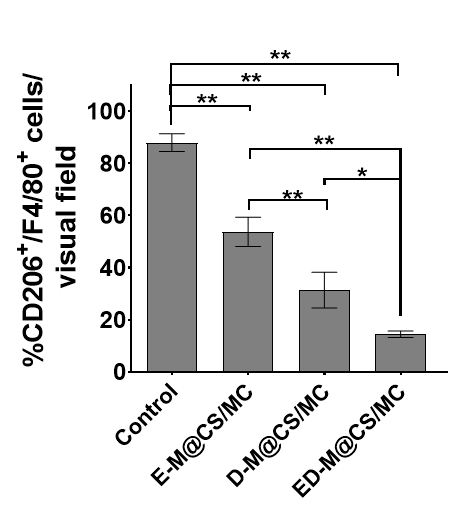

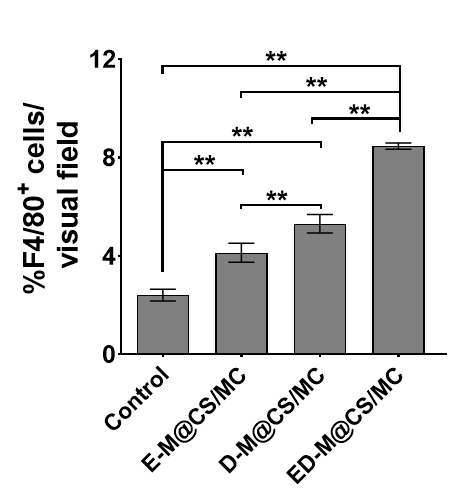


**Figure S12.** The proportion of M2 macrophages was analyzed by ImageJ (n = 3, Mean ± SD, * *P* < 0.05, ***P* < 0.01, ns, not significant)


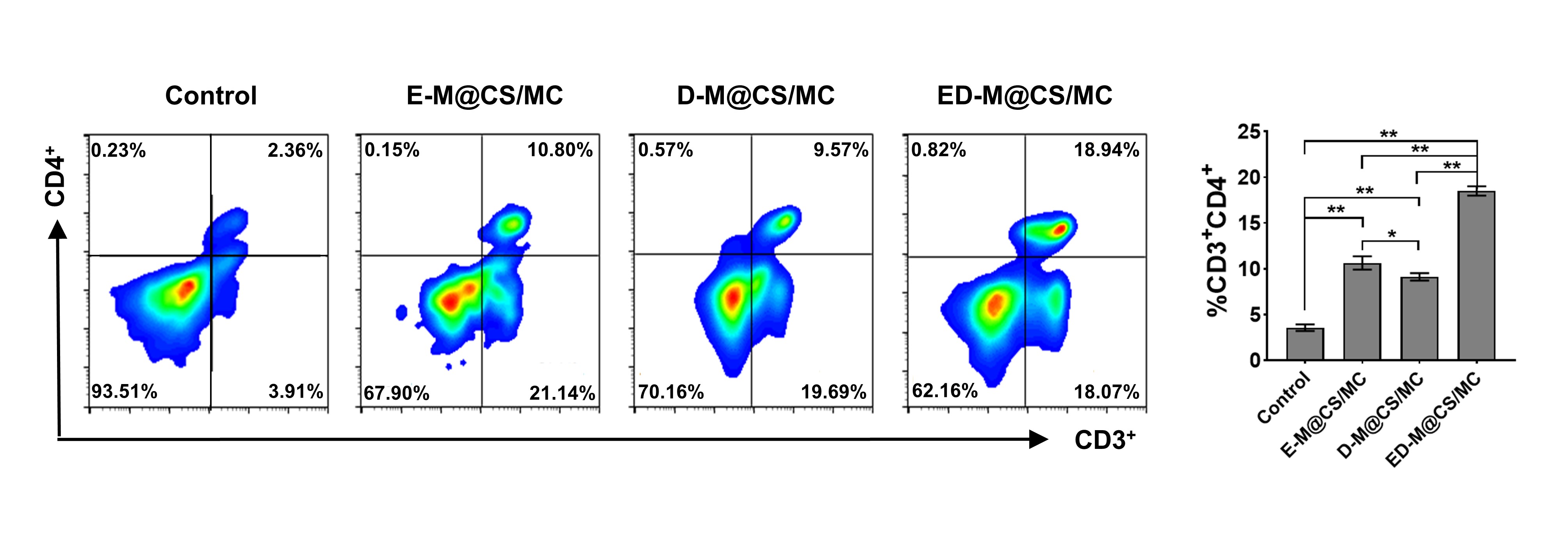


**Figure S13.** Representative flowcharts of CD3^+^CD4^+^ T cells and FCM analysis of CD3^+^CD4^+^ T cells in the tumor tissue (n = 3, Mean ± SD, **P*< 0.05, ***P*< 0.01)


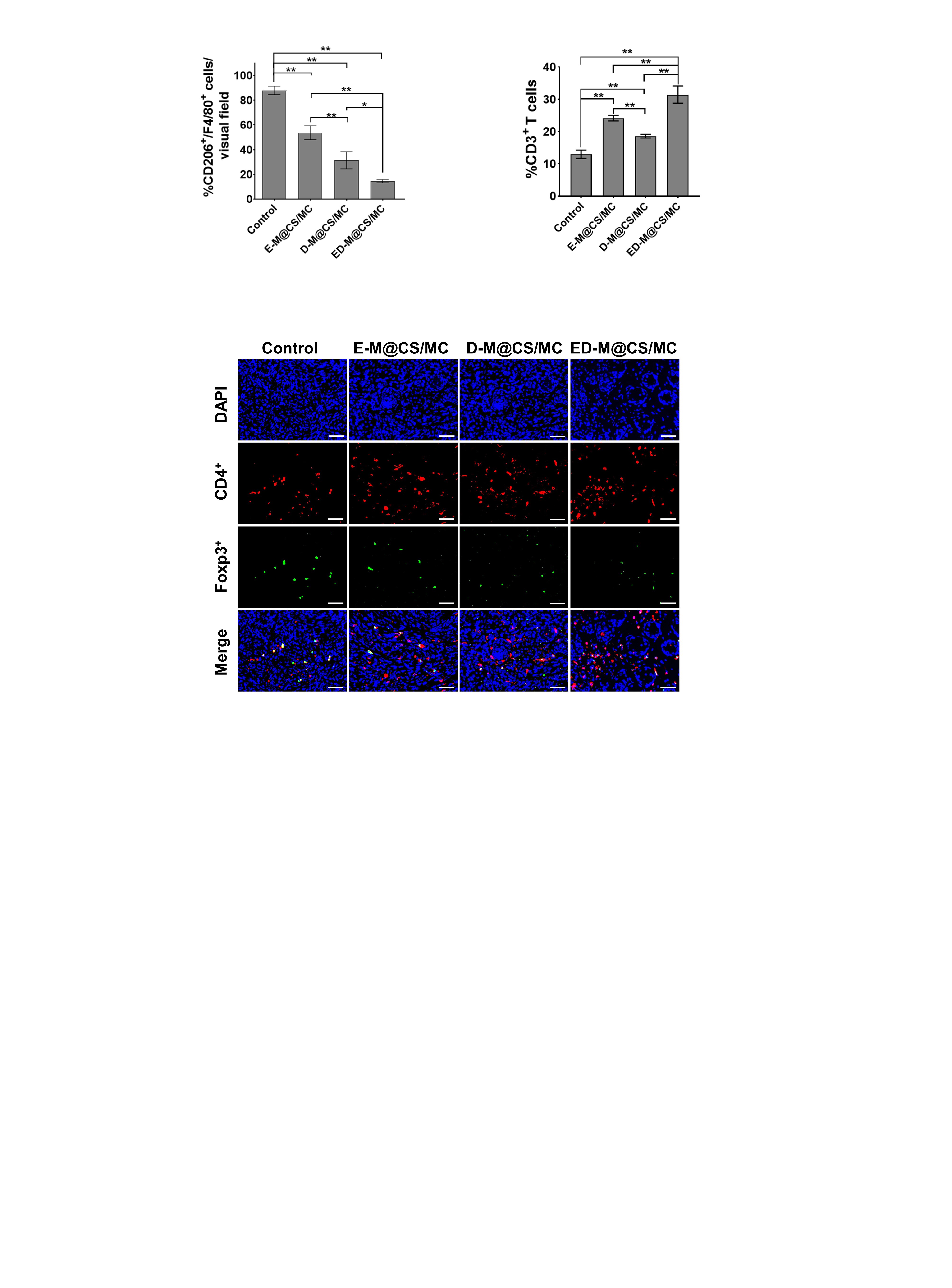


**Figure S14.** Representative immunofluorescence staining images of Treg (CD4^+^Foxp3^+^). Scale bar: 50 μm.


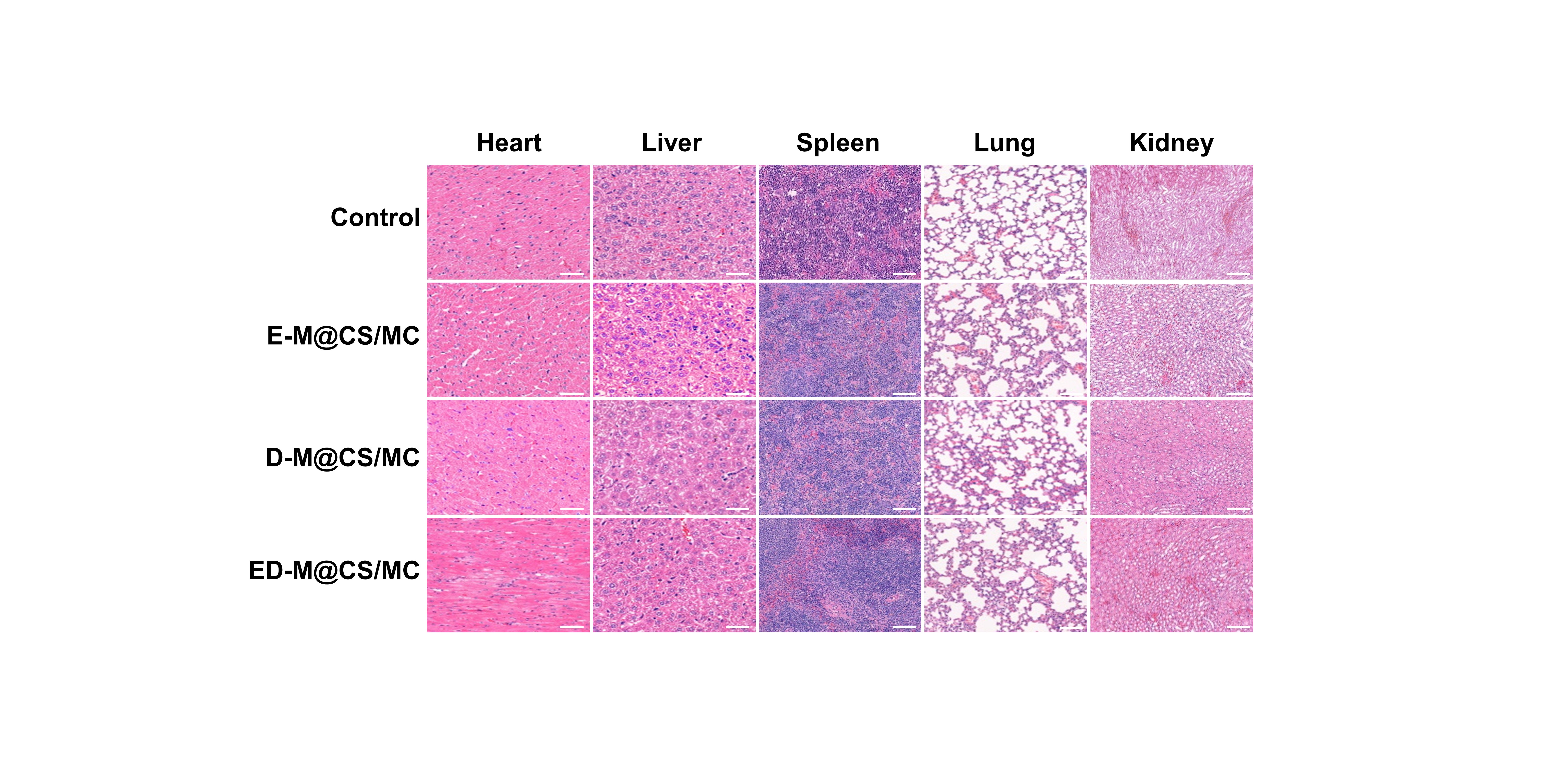


**Figure S15.** H&E staining of the major organs from the Kras^G12D^-engineering mice after different treatments. Scale bar: 100 μm.


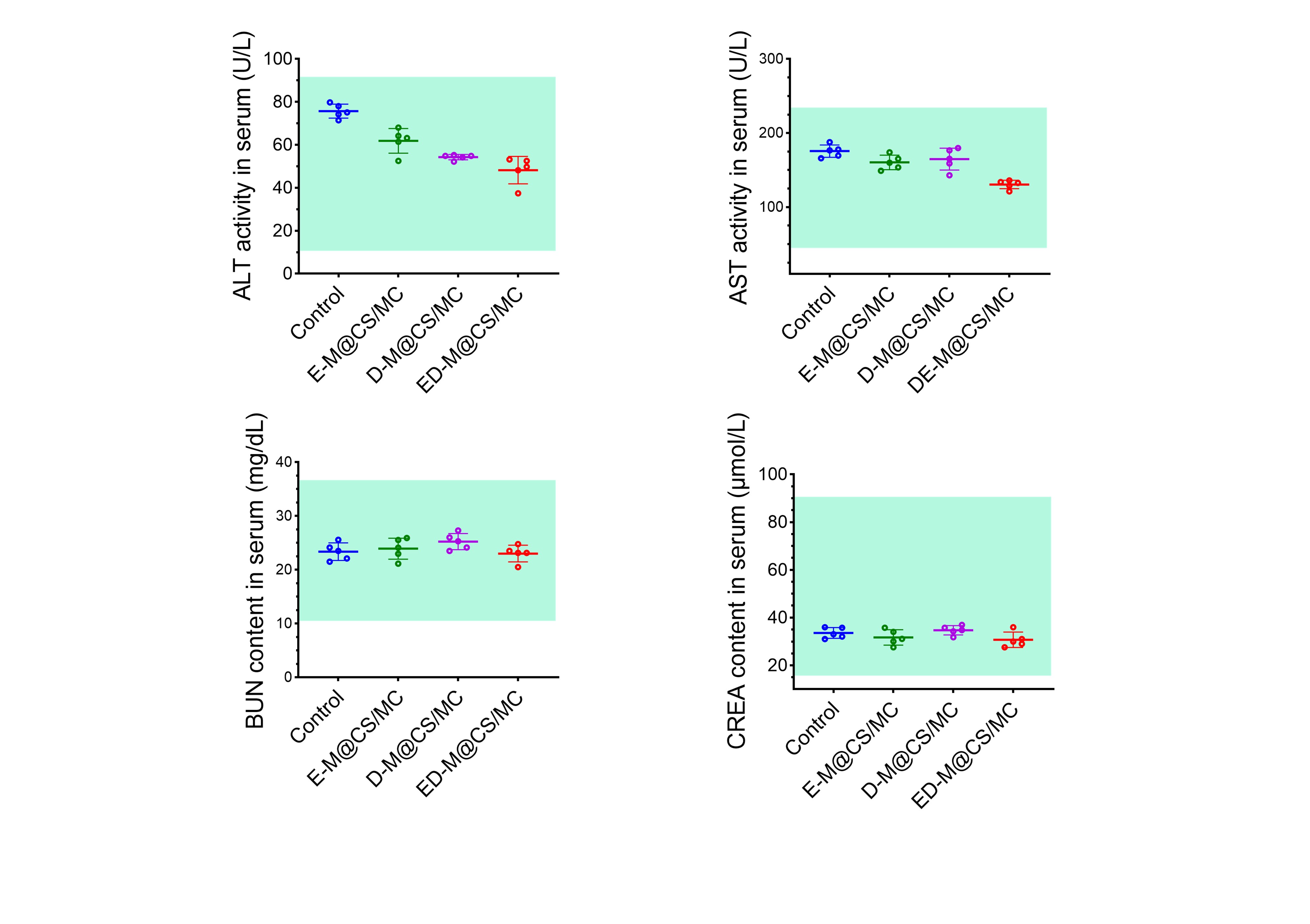


**Figure S16.** The serum concentrations of ALT, AST, BUN, and CREA in Kras^G12D^-engineering mice after different treatments (n = 5, Mean ± SD).

**
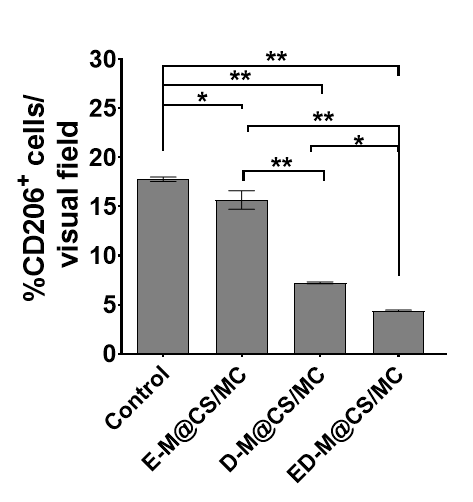
**

**Figure S17.** The proportion of M2 macrophages was analyzed by ImageJ (n = 3, Mean ± SD, * *P* < 0.05, ** *P* < 0.01, ns, not significant).

**
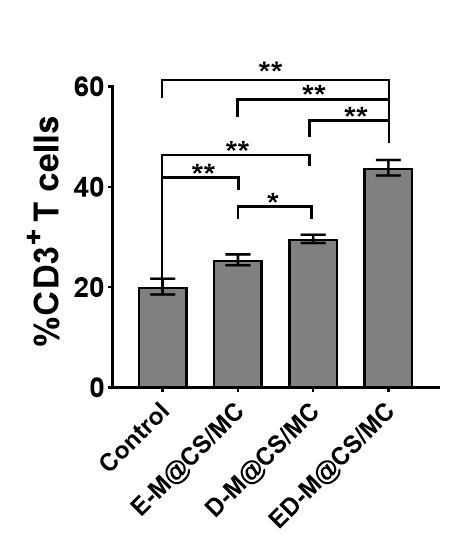
**

**Figure S18.** The proportion of M2 macrophages was analyzed by ImageJ (n = 3, Mean ± SD, * *P* < 0.05, ** *P* < 0.01).


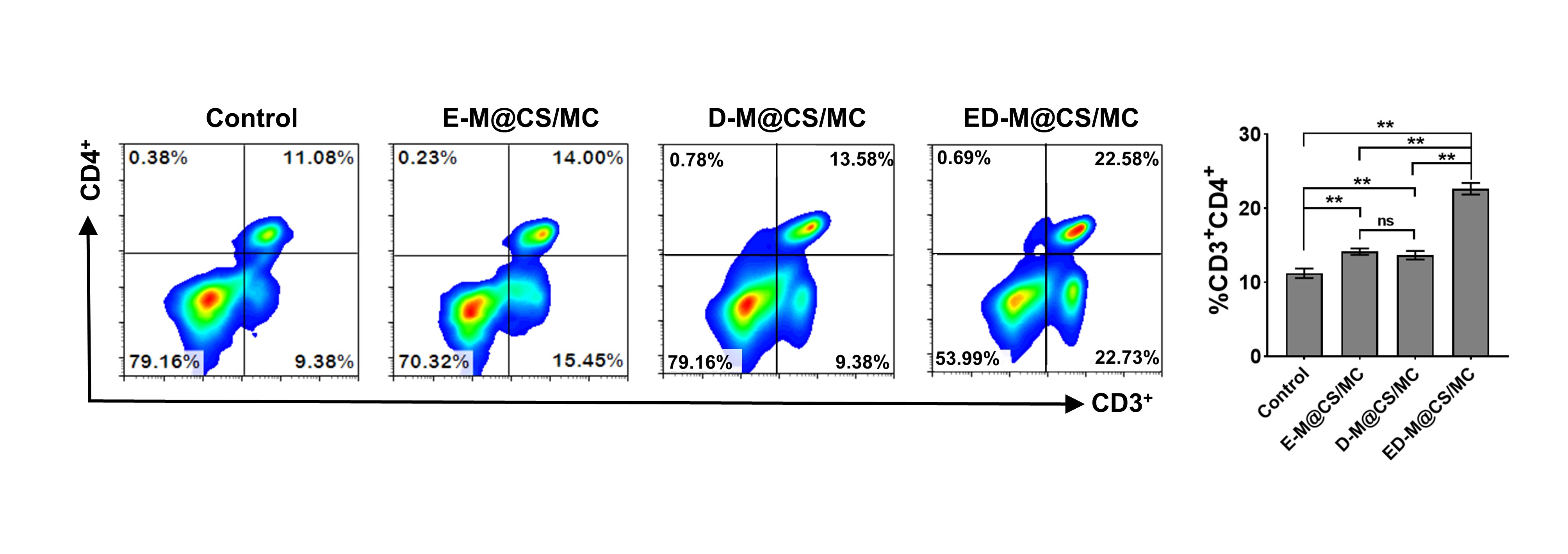


**Figure S19.** Representative flowcharts of CD3^+^CD4^+^ T cells and FCM analysis of CD3^+^CD4^+^ T cells in the tumor tissue (n = 3, Mean ± SD, **p* < 0.05, ***p* < 0.01).


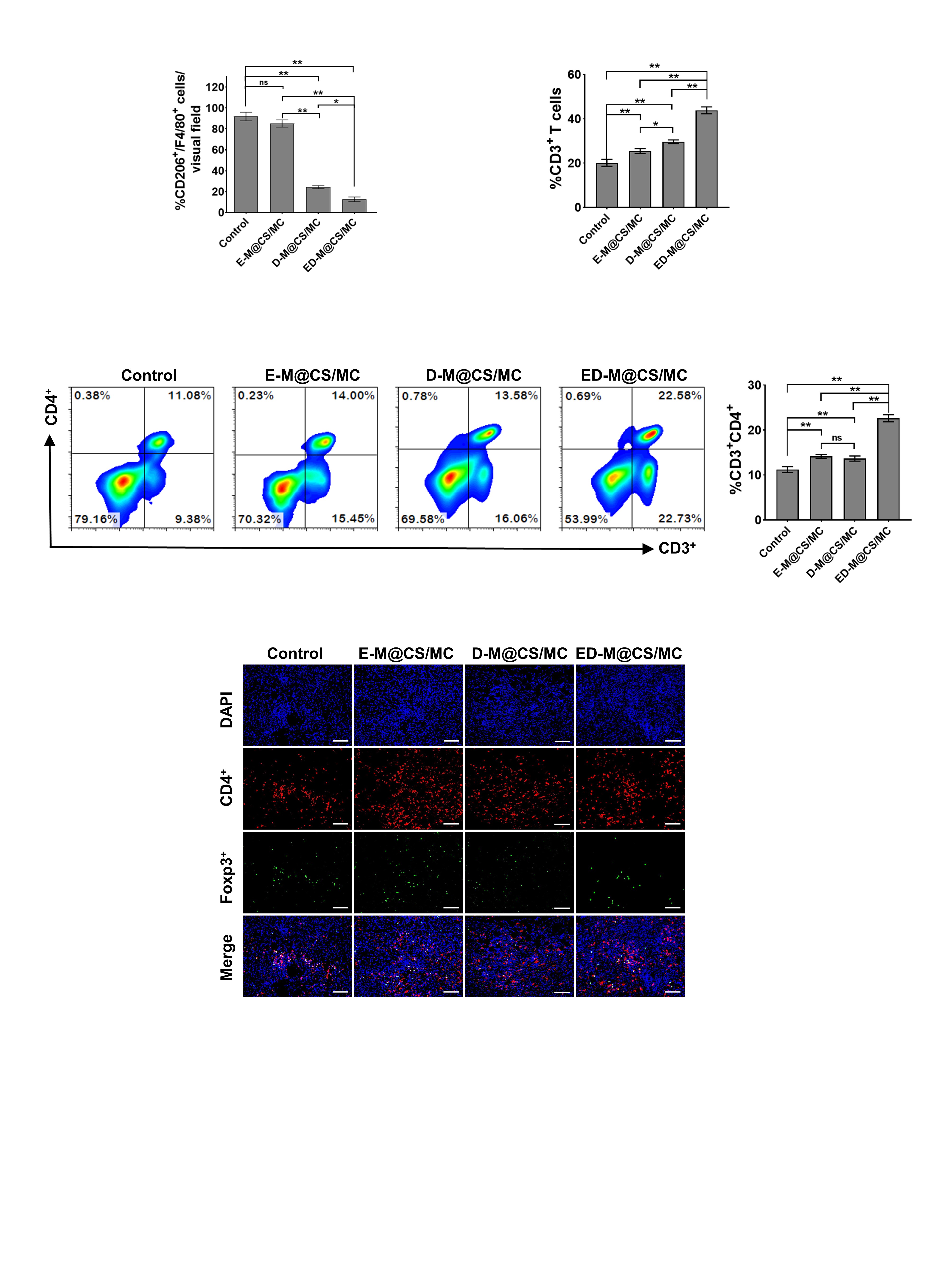


**Figure S20.** Representative immunofluorescence staining images of Treg (CD4^+^Foxp3^+^). Scale bar: 100 μm.

| **Table S1.** The gelation time of the CS/β-GP solution at 37°C | | |
| --- | --- | --- |
| Chitosan (w/v%) | β-GP (w/v%) | t_gel_ (min) |
| 2 | 2.0 | > 20 |
| 2 | 4.0 | > 20 |
| 2 | 5.0 | > 20 |
| 2 | 5.2 | 20 |
| 2 | 5.4 | 11 |
| 2 | 5.6 | 9 |
| 2 | 5.8 | 15 |

| **Table S2.** The gelation time of CS/MC solution at 37°C | | |
| --- | --- | --- |
| CS/β-GP (w/v%) | MC (w/v%) | t_gel_ (min) |
| 2.00/5.60 | 2.50 | 4 |
| 2.00/5.60 | 3.75 | 1 |
| 2.00/5.60 | 5.00 | 0.5 |

**Table S3.** The composition of each formulation

| Formulation | Erastin  (%, w/v) | Defactinib  (%, w/v) | mPEG_2000_-b-PDLLA_2000_ (%, w/v) | CS  (%, w/v) | β-GP  (%, w/v) | | | MC  (%, w/v) |
| --- | --- | --- | --- | --- | --- | --- | --- | --- |
| E-M@CS/MC | 0.056 | 0 | 0.625 | 2.00 | | 5.60 | 5.00 | |
| D-M@CS/MC | 0 | 0.054 | 0.625 | 2.00 | | 5.60 | 5.00 | |
| ED-M@CS/MC | 0.056 | 0.054 | 0.625 | 2.00 | | 5.60 | 5.00 | |
